# Tibetan Plateau Runoff and Evapotranspiration Dataset by an observation-constrained cryosphere-hydrology model

**DOI:** 10.1038/s41597-024-03623-3

**Published:** 2024-07-13

**Authors:** Xinfeng Fan, Lei Wang, Hu Liu, Deliang Chen, Lei Song, Yuanwei Wang, Jia Qi, Chenhao Chai, Ruishun Liu, Xiuping Li, Jing Zhou, Xiaoyu Guo, Junshui Long

**Affiliations:** 1 State Key Laboratory of Tibetan Plateau Earth System, Environment and Resources, Institute of Tibetan Plateau Research, Chinese Academy of Sciences, Beijing, 100101 China; 2 University of Chinese Academy of Sciences, Beijing, 100049 China; 3 Regional Climate Group, Department of Earth Sciences, University of Gothenburg, Gothenburg, 40530 Sweden; 4 PIESAT Information Technology Co., Ltd, Beijing, People’s Republic of China; 5 School of Geographical Sciences, Nanjing University of Information Science and Technology, Nanjing, 210044 China; 6 Binhai New Area Meteorological Office of Tianjin, Tianjin, 300450 China; 7 School of Surveying and land information Engineering, Henan Polytechnic University, Jiaozuo, 454003 China

**Keywords:** Hydrology, Cryospheric science

## Abstract

Runoff and evapotranspiration (ET) are pivotal constituents of the water, energy, and carbon cycles. This research presents a 5-km monthly gridded runoff and ET dataset for 1998–2017, encompassing seven headwaters of Tibetan Plateau rivers (Yellow, Yangtze, Mekong, Salween, Brahmaputra, Ganges, and Indus) (hereinafter TPRED). The dataset was generated using the advanced cryosphere-hydrology model WEB-DHM, yielding a Nash coefficient ranging from 0.77 to 0.93 when compared to the observed discharges. The findings indicate that TPRED’s monthly runoff notably outperforms existing datasets in capturing hydrological patterns, as evidenced by robust metrics such as the correlation coefficient (CC) (0.944–0.995), Bias (−0.68-0.53), and Root Mean Square Error (5.50–15.59 mm). Additionally, TPRED’s monthly ET estimates closely align with expected seasonal fluctuations, as reflected by a CC ranging from 0.94 to 0.98 when contrasted with alternative ET products. Furthermore, TPRED’s annual values exhibit commendable concordance with operational products across multiple dimensions. Ultimately, the TPRED will have great application on hydrometeorology, carbon transport, water management, hydrological modeling, and sustainable development of water resources.

## Background & Summary

Runoff and evapotranspiration (ET) are principal elements of the water cycle, markedly influencing the interchange of water, energy, and carbon among the terrestrial biosphere, hydrosphere, and atmosphere^1–3^. Within the global hydrological cycle, surface precipitation is partitioned into runoff (approximately one-third) and ET (about two-thirds). Notably, the ET consumes more than half of the total global absorbed solar radiation^4–7^. The Tibetan Plateau (TP), often referred to as the “Asian Water Tower”, is the source of numerous large rivers. It plays a pivotal role in supplying water to both ecological environments and human societies in downstream areas^8–10^. In the context of global change, the TP serves as a potential “tipping point” in the Earth system, exerting substantial regional and global influences^11–13^. Currently, the TP is currently experiencing significant cryosphere ablation owing to regionally amplified warming, which has a marked impact on water, energy, and carbon cycles, and lead to increased occurrences of extreme hydrological events and uncertainties in the carbon budget^14–21^.

To date, generating reliable runoff and ET datasets for high-altitude river basins across the TP has been challenging, hindered by the scarcity of quality-controlled observational data^22–28^. This lack is exacerbated by harsh environmental conditions, infrastructural limitations, and restrictive data-sharing policies, particularly for transboundary river data^29^. Furthermore, the limited availability of observed data present challenges in accurately depicting the large-scale spatial heterogeneity of hydrometeorology in the high-mountain basins.

Addressing these challenges necessitates the creation of large-scale gridded runoff and ET products across the TP by integrating sparse ground observations with extensive remote sensing and reanalysis datasets. These could achieve through physics-based hydrological models or data-driven machine learning and multi-source data fusion techniques. The water input in the TP’ water balance is supplied not only by precipitation but also by the storage and regulation from glaciers, snow, and permafrost^30–32^; however, numerous current research approaches of gridded runoff and ET products do not adequately account for cryosphere-hydrological processes^25–28,33–43^.

Given the TP’s significant elevation gradients and diverse topography, the relatively coarse spatial resolution of existing products fails to accurately capture fine variations in runoff and ET, particularly within montane river valleys^11^. High-resolution hydrological modeling shows significant advantages in detailed exploration of complex topographic areas, thereby markedly improving the accuracy of hydrological simulations compared to those employing lower resolutions^22,44–47^.

This study aims to generate a high-quality 5-km spatial resolution monthly gridded dataset of runoff and ET for the headwater regions of major rivers across the TP, referred to as TPRED. These rivers include the Yellow, Yangtze, Mekong, Salween, Brahmaputra, Ganges, and Indus rivers, covering the TP from east to west, spanning 1998 to 2017. The TPRED was generated using the advanced cryosphere-hydrology WEB-DHM model, which was constrained by valuable observed discharge at the outlet of each headwater. This dataset is positioned to significantly contribute to various research fields including hydrology, meteorology, water-carbon cycle studies, water resource management, and the improvement of hydrological model accuracy, thereby advancing the goals of sustainable development for water resources across the TP.

## Methods

### Study area

The Tibetan Plateau (TP) constitutes the third-largest cryospheric area globally, following only Antarctica and the Arctic^48,49^. At an average altitude of over 4,500 meters, the TP is recognized as the highest plateau on Earth. The TP receives its water supply from both monsoon and westerly, alongside the meltwater from glaciers, snow and frozen soil, making it a crucial water source for numerous major rivers that extend from High Mountain Asia to the densely populated lowlands^8–10,43^.

**Fig. 1:**
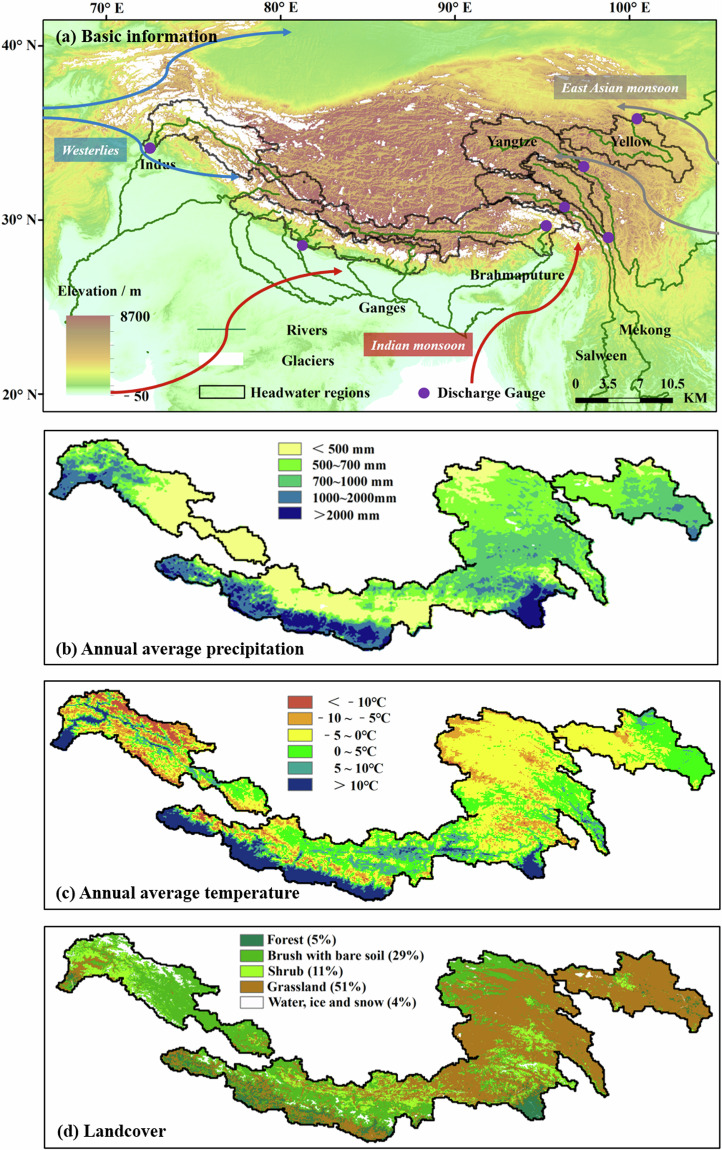
Headwater regions of seven major rivers across the TP. (**a**) The hydrological observation stations located at the outlet of each headwater region are Tangnaihai (Yellow River), Zhimenda (Yangtze River), Changdu (Mekong River), Jiayuqiao (Salween River), Nuxia (Brahmaputra River), Karnali (Ganges River), and Besham (Indus River). (**b,** **c**) Annual average precipitation, temperature and landcover types and proportion across seven headwaters. See Table 2 for the sources of these data.

This study focuses on the headwaters of seven major exorheic rivers across the TP from east to west: the Yellow, Yangtze, Mekong, Salween, Brahmaputra, Ganges, and Indus rivers (Fig. 1a). These headwaters exhibit diverse hydrometeorological characteristics, shaped by their individual responses to climatic and environmental changes^50,51^. Details regarding these river headwaters are presented in Table 1.

**Table 1:** Basic characteristics of seven headwater regions across the TP.

Climate zone	*Transition region*		*Indian Monsoon dominated region*				*Westerlies dominated region*
River headwater	Yellow	Yangtze	Mekong	Salween	Brahmaputra	Ganges	Indus
**Area / 10**^**4**^ **km**^**2**^	13.2	14.9	7.7	7.9	26.2	18.8	20.9
**Glacier coverage / %**	0.1	0.9	0.5	1.6	6.1	6.3	9.8
**Annual runoff / mm**	216	140	234	316	683	1,011	553
**Annual ET / mm**	466	298	563	339	348	530	375
**Annual precipitation / mm**	494	432	802	837	642	1,430	756

The specific values are obtained from the inputs and outputs of the validated WEB-DHM model.

High elevations lead to almost half of the region experiencing average annual temperature below freezing, notably in the headwaters of the Indus and Yangtze (Fig. 1b and Table 2). These areas have shown a sharp increase in temperatures over recent decades, with warming rates significantly exceeding those of comparable latitudes^14^. Precipitation distribution varies significantly across these headwaters, with higher rainfall observed in the southern regions of the Ganges and the southeastern areas of the Brahmaputra, often exceeding 1,000 mm annually (Fig. 1c and Table 2). Climate change has further altered precipitation patterns, increasing their irregularity and complexity^15^.

**Table 2:** Datasets used in model input and evaluation.

Data type	Yellow	Yangtze	Mekong	Salween		Brahmaputra	Ganges	Indus	
Precipitation	CMA (https://data.cma.cn/en)	CMFD^80^	TPFMD^81^	ERA5^33^		IGSR^82^	TPFMD	ERA5-land^34^	
Other six meteorological variables				CMFD		CMFD		GLDAS^83^	
Leaf area index (LAI)	GLASS^84^								
Fraction of photosynthetically active radiation (FPAR)									
Glacier data (glacier extent, debris-cover)	—		Second Glacier Inventory of China^85–88^				ICIMOD^71^		
Digital elevation model (DEM)	SRTM (http://srtm.csi.cgiar.org/)								
Land use	USGS (http://edc2.usgs.gov/glcc/glcc.php)								
Soil organic carbon	SRIC-World Soil Information (https://soilgrids.org/)								
Soil data (soil type, soil depth)	FAO (http://www.fao.org/geonetwork/srv/en/main.home)								
*In situ* discharge observations	Ministry of Water Resources of China (http://www.mwr.gov.cn/english/)				TP-River project^29^				WAPDA^29^
Land surface temperature	MODIS^89^								
Snow data (snow depth, snow cover)	SSM/I snow depth (https://data.tpdc.ac.cn/zh-hans/data/47acf141-2c86-4e1f-8f68-036ae57d268e)		M*D10A1GL06 snow cover^90^						

For a comprehensive exploration of these datasets, please refer to the Supplement “Part 1.1”.

The distinct climatic and environmental conditions across the TP support a diverse ecosystem with complex vegetation patterns. Grasslands dominate half of the landscape across these headwaters (Fig. 1d and Table 2). A warming and moistening of the climate has induced a visible “greening” effect on the vegetation^52^. Moreover, the changing climate and surface environment have significantly influenced the regional hydrologic cycle^53^, as evidenced by the spatiotemporal variations in runoff and ET^54,55^.

### Cryosphere-hydrology model

The TP serves as a key area where the atmosphere, hydrosphere, biosphere, lithosphere, and cryosphere interact, with the hydrological cycle playing a central role in these interactions^44,56–58^. However, many existing studies exploring hydrological processes on the TP have been hindered by methodological constraints that do not fully consider these multi-sphere interactions. Considering these limitations, Wang *et al*.^59^ coupled a biosphere land surface model (SiB2) with a geomorphology-based hydrological model (GBHM) to develop the Water and Energy Budget-based Distributed Hydrological Model (WEB-DHM)^59^. This model considers water, energy, and CO_2_ fluxes within soil-vegetation-atmosphere systems at the grid scale, which not only incorporates energy conservation in land surface processes, but also delivers detailed representations of hydrophysical phenomena at high spatial resolutions^59–62^. The structure of the model at the grid scale is shown in Fig. S1. In addition, the WEB-DHM model has demonstrated its effectiveness in elucidating effects of cryosphere change on hydrological processes.

For snow simulation, the WEB-DHM incorporates an advanced enthalpy-based snow processes, employing the three-layer energy-balance snow parameterization from Simplified Simple Biosphere 3 (SSib3) and the prognostic albedo scheme from the Biosphere–Atmosphere Transfer Scheme (BATS)^60,62–65^. This module separates the snowpack into layers at each grid cell, with snow depths exceeding 5 cm being categorized into three layers; shallower depths, and those on canopy, are treated as a single layer (Fig. S2). This module considers the energy exchange between snow layers and the impact of solar radiation incidence angle and snow age on albedo. To sum up, this model could grant a meticulous depiction of snow physics, inclusive of phase transitions, compaction, albedo variations, temperature profiles, and meltwater runoff for each layer^60,62–65^.

Simultaneously, the model’s empirical frozen ground parameterization has been enhanced, encapsulating frozen ground dynamics through a hydrothermal transfer parameterization using the Johansen thermal conductivity framework^66^ (Fig. S2). By employing enthalpy rather than temperature in energy balance calculations, this approach reduces uncertainties associated with phase change latent heat release and improves model stability^61,67,68^. As a result, the model effectively simulates freeze-thaw cycles at fine scales and basin-scale hydrological processes, while appropriately accounting for subgrid topographic variations^67^.

The WEB-DHM model also integrates an energy balance-oriented glacier module to address both clean and debris-covered glaciers, thus augmenting the model’s capabilities for glacier processing^69^. For clean glaciers, a similar three-layer water- and energy-balance scheme is used, whereas a single-level scheme that accounts for debris influence on energy absorption is employed for debris-covered glaciers.

### Water balance method

Given the challenge of directly measuring ET at the basin scale, WEB-DHM ET data were indirectly validated against the residual of precipitation (*P*) minus observed runoff (*R*_*obs*_) and terrestrial water storage changes measured by the Gravity Recovery and Climate Experiment (GRACE) satellite (*ΔS*_*GRACE*_) as the reference value^42^. However, this water balance approach dependent on the GRACE satellite is only applicable to very large regions, and cannot account for glacier-related changes in complex mountain regions (e.g., Ganges and Indus basins). Consequently, this method was only employed to evaluate the simulated ET for the Yellow, Yangtze, and Brahmaputra (upstream of the Nuxia Station) basins, where the glacier coverage is minimal and the basin area is large.

### Performance metrics

A suite of metrics was utilized to assess the performance of the model and reliability of various products; these included the *Nash* coefficient^70^, Bias, Root Mean Square Error (*RMSE*), and Correlation Coefficient (*CC*)^71^. These metrics are computed as follows:

1 $${Nas}h=1-\frac{\mathop{\sum }\limits_{i=1}^{n}{\left({X}_{i}-{{ref}}_{i}\right)}^{2}}{\mathop{\sum }\limits_{i=1}^{n}{\left({{ref}}_{i}-\overline{{ref}}\right)}^{2}}$$

2 $${Bias}=\frac{\mathop{\sum }\limits_{i=1}^{n}\left({X}_{i}-{{ref}}_{i}\right)}{\mathop{\sum }\limits_{i=1}^{n}{{ref}}_{i}}\times 100 \% $$

3 $${RMSE}=\sqrt{\frac{\mathop{\sum }\limits_{i=1}^{n}{\left({X}_{i}-{{ref}}_{i}\right)}^{2}}{n}}$$

4 $${CC}=\frac{\mathop{\sum }\limits_{i=1}^{n}\left({X}_{i}-\bar{X}\right)\left({{ref}}_{i}-\overline{{ref}}\right)}{\sqrt{\mathop{\sum }\limits_{i=1}^{n}{\left({X}_{i}-\bar{X}\right)}^{2}}\sqrt{\mathop{\sum }\limits_{i=1}^{n}{\left({{ref}}_{i}-\overline{{ref}}\right)}^{2}}}$$

here, ${{\boldsymbol{ref}}}_{{\boldsymbol{i}}}$ represents the reference value at the ***i*** moment, such as observed flow or ET calculated using the water balance method; ***X*** is the target variable being evaluated, and ***n*** is the number of samples; $\overline{{\boldsymbol{ref}}}$ and $\bar{{\boldsymbol{X}}}$ represent the mean values of the reference sequence ${{\boldsymbol{ref}}}_{{\boldsymbol{i}}}$ and target sequence ${{\boldsymbol{X}}}_{{\boldsymbol{i}}}$, respectively. The closer the values of *Nash* and *CC* are to 1, the higher the reliability of the target variable. Conversely, the closer the values of *RMSE* and *Bias* are to 0, the higher the reliability of the target variable.

### Datasets used in model input and evaluation

**Fig. 2:**
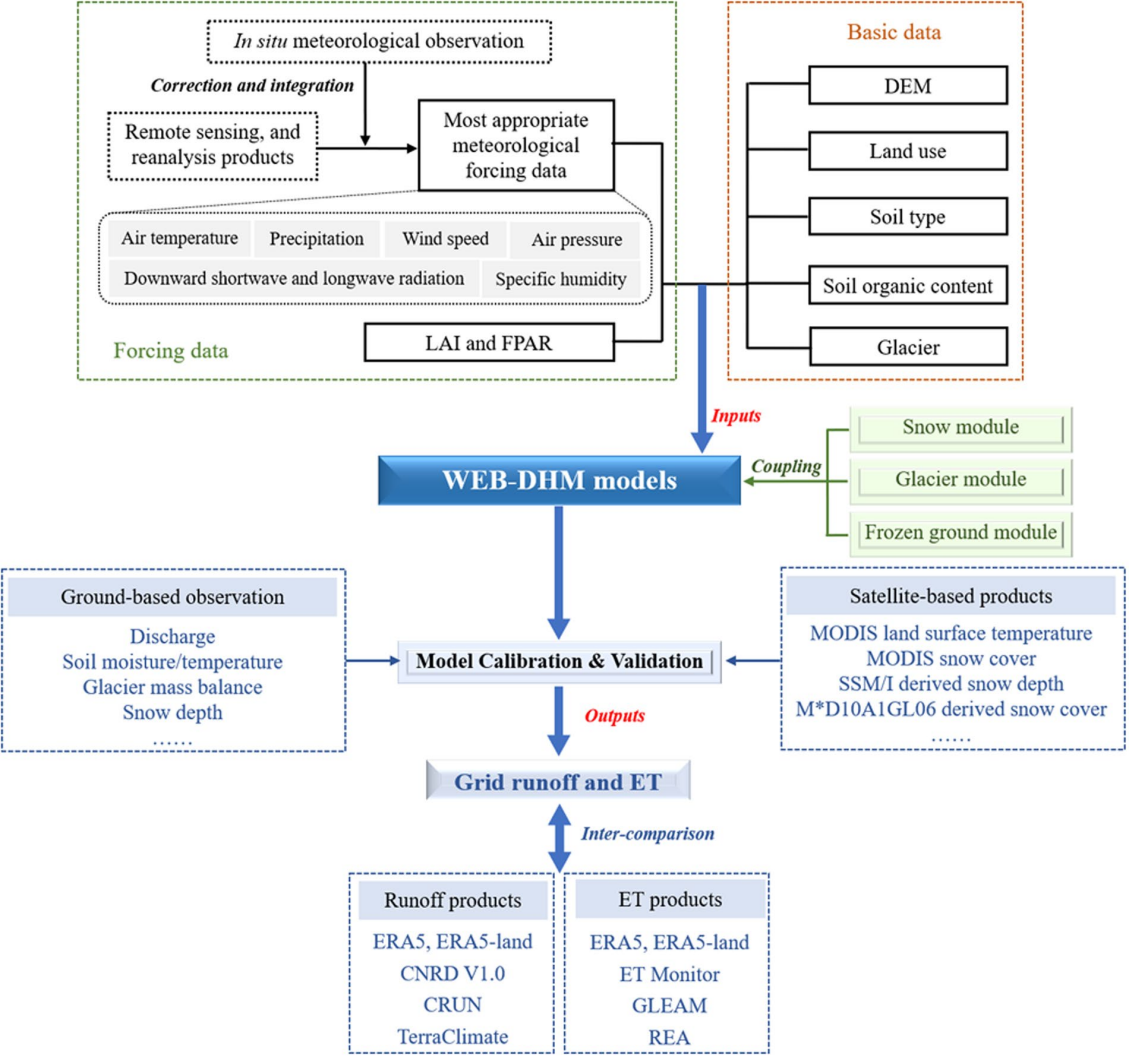
Flowchart of this study.

The WEB-DHM model incorporated *in situ* observations, reliable remote sensing, and reanalysis data as input. These datasets comprised observed daily discharge, gridded meteorological products, vegetation dynamic products, DEM, as well as detailed maps of land cover, soil, and glacier. Additionally, diverse satellite-based products such as land surface temperature and snow depth were utilized to validate the model’s multiple outputs. Specific datasets for each headwater model are listed in Table 2 and illustrated in Fig. 2.

### Data products for cross-comparison with TPRED

In recent decades, there has been a marked increase in runoff and ET products, each characterized by distinct input data, spatiotemporal resolutions, and modeling methodologies. While *in situ* measurements provide exceptional accuracy, remote sensing, model simulations, and data fusion techniques also contribute significantly, despite their inherent uncertainties^23,25,29,72–75^. These uncertainties stem from global discrepancies in gauge coverage, difficulties in accurately representing hydrophysical processes within remote sensing or machine learning frameworks, and the constraints about model performance or the quality of model inputs^37,38,41,76–78^. A detailed summary of limitations associated with numerous state-of-the-art runoff and ET products in the TP has been conducted; please refer to the Supplement “Part 1.2” for more information.

Therefore, it is crucial to meticulously evaluate the appropriateness of different data products for specific applications within the study region. This study presents a comprehensive comparison of the TPRED dataset with current gridded runoff and ET datasets, including runoff products from ERA5, ERA5-land, CNRD V1.0, CRUN, TerraClimate, and ET products from ERA5, ERA5-land, REA, ET Monitor, and GLEAM, as detailed in Table 3. Table 3 highlights the recurring deficiencies in these existing datasets, such as insufficient observations within the TP, relatively coarse spatial resolution, and frequent omission of critical cryosphere-hydrophysical processes.

**Table 3:** Datasets or products for cross-comparisons with TPRED.

Variable	Dataset	Method	*In situ* observations for calibration or validation	Consideration of cryosphere hydrologic processes such as snow, glacier and frozen soil	Input data	Spatial resolution	Selected period	Download link
Runoff and ET	TPERD	WEB-DHM cryosphere-hydrology model	Seven hydrological sites	Snow, glacier and frozen soil	Climate, soil, vegetation, land use, glacier, topographical data	5 km	1998–2017	https://zenodo.org/records/10060590
	ERA5^91–93^	HTESSEL Land surface model	None	Snow	Climate data	0.25°	1998–2017	https://cds.climate.copernicus.eu/cdsapp#!/dataset/reanalysis-era5-single-levels-monthly-means?tab=form
	ERA5-land^34^	CHTESSEL Land surface model	None	Snow, glacier and frozen soil	Climate data	0.1°	1998–2017	https://cds.climate.copernicus.eu/cdsapp#!/dataset/reanalysis-era5-land-monthly-means?tab=overview
Runoff	CNRD V1.0^26^	VIC hydrological model	Seven hydrological sites	None	Climate, soil, vegetation, topographical data	0.25°	1998–2017	https://data.tpdc.ac.cn/en/data/8b6a12c7-c8f9-465a-b449-852fbff51853/
	CRUN^25^	Machine learning algorithm	Three hydrological sites	None	Climate data	0.5°	1998–2014	https://figshare.com/articles/dataset/GRUN_Global_Runoff_Reconstruction/9228176
	TerraClimate^78^	Water-balance model	None	Snow	Climate data	1/24°	1998–2015	https://data.nkn.uidaho.edu/dataset/monthly-climate-and-climatic-water-balance-global-terrestrial-surfaces-1958-2015
ET	REA^43^	Ensemble averaging method	One flux tower site	None	ERA5, GLDAS2, and MERRA-2 ET data	0.25°	1998–2017	https://zenodo.org/records/4595941
	ET Monitor^40^	ET Monitor model	Two flux tower sites	Snow	Climate, soil moisture, vegetation, land use data	1 km	2000–2017	https://data.tpdc.ac.cn/en/data/c284bd88-7694-4577-9cbb-02684bd940ff
	GLEAM^39^	Global Land Evaporation Amsterdam Model	None	Snow	Climate, soil moisture, soil properties data	0.25°	1998–2017	https://www.gleam.eu/

## Data Records

The TPRED has been uploaded to the *Zenodo* platform and is publicly accessible via https://zenodo.org/records/10060590 or 10.5281/zenodo.10060590^79^. The time range is 1998–2017 at a monthly scale, the spatial resolution is 5 km × 5 km, and the unit is mm/month. For the convenience of users, this data is stored in TIF format, with each combination of different watersheds forming a separate file. Each file contains two variables: either runoff or ET.

## Technical Validation

### Calibration and validation of WEB-DHM models

We conducted model calibration and validation utilizing both ground-based and satellite-based observations, with a primary focus on observed daily discharge (Fig. 2). Model calibration involves adjusting saturated hydraulic conductivity (*K*_*surface*_), soil anisotropy ratio (*anik*), two van Genuchten parameters (*α* and *n*) and so on. Among these, *K*_*surface*_ and *anik* are identified as the most sensitive parameters influencing streamflow simulations within the WEB-DHM. *K*_*surface*_ primarily modulates peak flow, whereas *anik* impacts both peak and recession flows^60,66^. The *α* and *n* adjust the soil hydraulic function, influencing water transport within the soil and consequently affecting the flood hydrograph. Furthermore, a sensitivity study for these model parameters in the WEB-DHM has already been provided (https://hess.copernicus.org/preprints/6/C3455/2010/hessd-6-C3455-2010-supplement.pdf)^60^.

Building on the calibration result, the optimized parameters are subsequently employed during the validation phase. Table 4 presents the metrics (e.g., *Nash* and *Bias*) for the model performance during the calibration and validation periods, demonstrating that the WEB-DHM produces satisfactory results across different headwaters.

**Table 4:** The model assessment during the calibration and validation periods for each of the study headwaters.

River headwater	Hydrological station	Calibration		Validation	
		Period	Index	Period	Index
Yellow^61,67,68^	*Tangnaihai*	1981–1985	*Nash* = 0.81; *Bias* = −4.12%	1986–2019	*Nash* = 0.74; *Bias* = 5.50%
Yangtze^29,94^	*Zhimenda*	1981–1985	*Nash* = 0.71; *Bias* = 1.50%	1986–2016	*Nash* = 0.62; *Bias* = −2.90%
Mekong	*Changdu*	1998–2004	*Nash* = 0.68; *Bias* = 5.42%	2005–2014	*Nash* = 0.66; *Bias* = −12.61%
Salween^70^	*Jiayuqiao*	1981–1983	*Nash* = 0.70; *Bias* = 3.18%	1984–1987	*Nash* = 0.79; *Bias* = 3.29%
Brahmaputra^87,88,95^	*Nuxia*	2012–2016	*Nash* = 0.62; *Bias* = 0.11%	1998–2011; 2017-2018	*Nash* = 0.66; *Bias* = −2.89%
Ganges	*Karnali*	2001–2004	*Nash* = 0.81; *Bias* = −12.8%	1981–2000; 2005–2019	*Nash* = 0.63; *Bias* = −17.12%
Indus^71^	*Besham*	2001–2004	*Nash* = 0.86; *Bias* = 14.50%	2005–2020	*Nash* = 0.87; *Bias* = −3.00%
	*Gligit*	—	—		*Nash* = 0.68; *Bias* = −16.40%
	*Hunza*	—	—		*Nash* = 0.84; *Bias* = 12.80%
	*Shyok*	—	—		*Nash* = 0.81; *Bias* = 28.90%
	*Astore*	—	—		*Nash* = 0.72; *Bias* = −15.20%

### Evaluation of the WEB-DHM outcomes against the observed reference

**Fig. 3:**
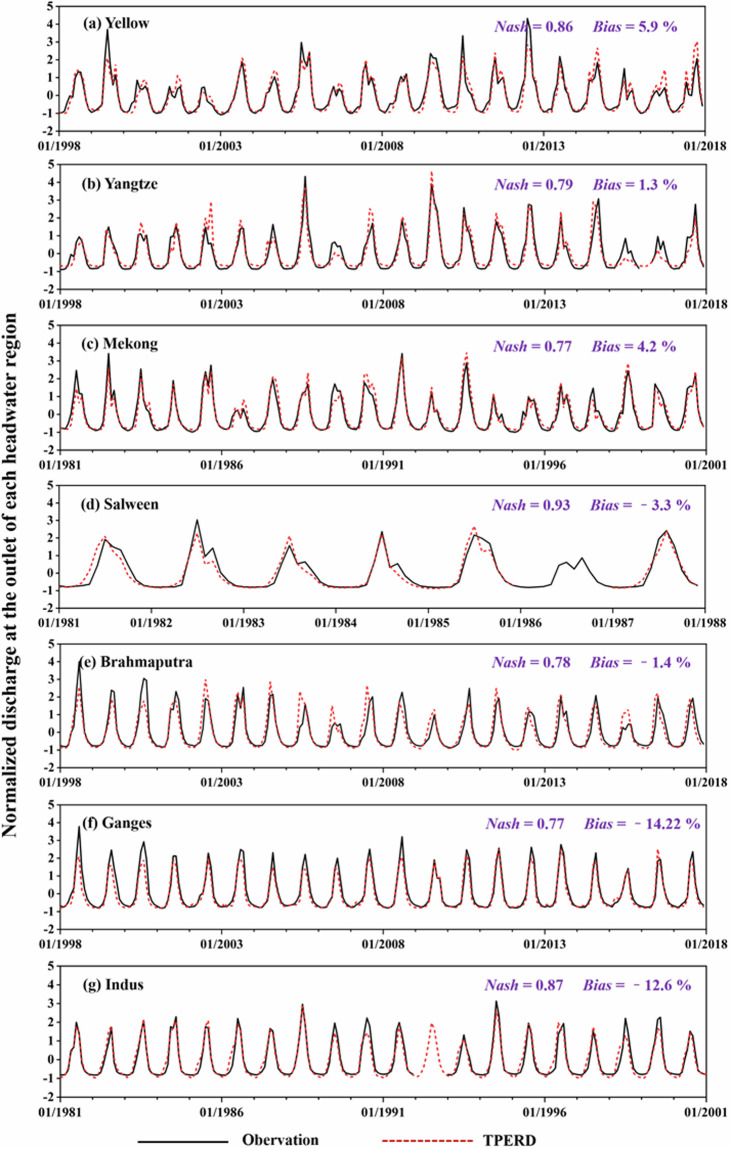
Normalized observed and simulated discharge at the outlet of each headwater region across the TP. The *Z-score* method is used to standardize the data series^96^.

The rigorous assessment of outcomes of the WEB-DHM model was conducted across different basins. Figure 3 shows a comparison between the model-simulated monthly streamflow and the observed flow at the outlet of each headwater region, utilizing *Nash* and *Bias* scores as evaluation metrics. The WEB-DHM estimations of monthly river flow demonstrated satisfactory agreement with the observational data across seven headwaters. This is evidenced by *Nash* values spanning the range 0.77–0.93, and *Bias* values oscillating between −14.2% and 5.9%. The simulated discharge adeptly captured the temporal pattern of river flow, closely mirroring the peaks and troughs of observed streamflow in terms of magnitude and timing.

**Fig. 4:**
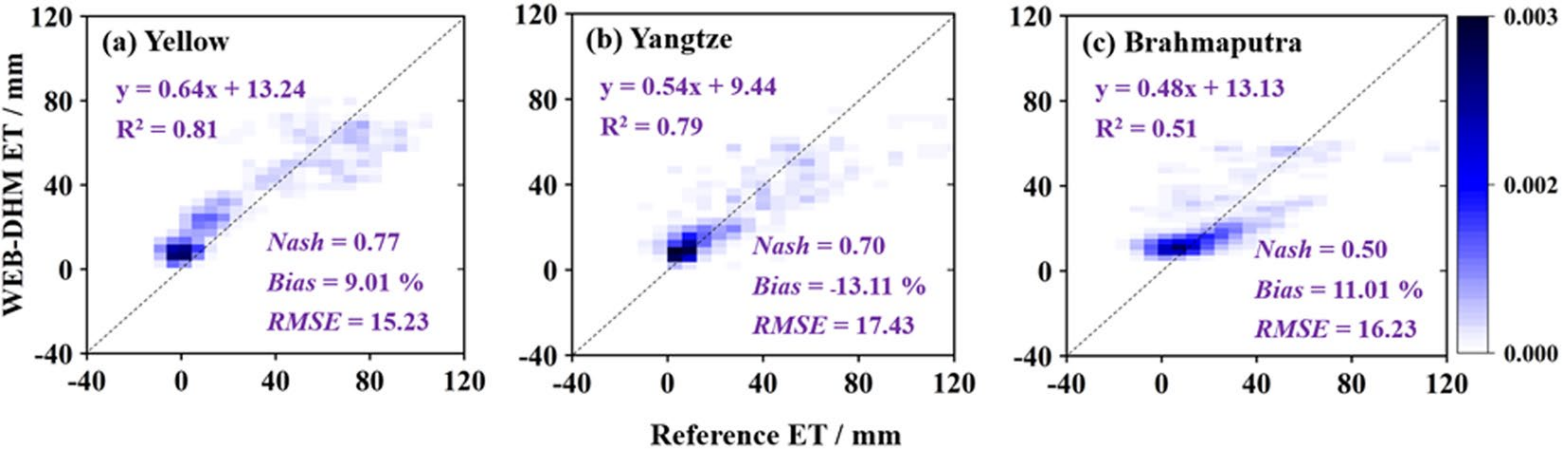
Comparison of the TPRED ET with the monthly reference ET. Reference ET was obtained from the water balance approach (=*P* – *R*_*obs*_ – Δ*S*_*GRACE*_), averaged over the Yellow, Yangtze, and Brahmaputra headwater regions. The right bar signifies the density scale, where a higher value corresponds to an increased concentration.

For the TPRED ET simulations, the model demonstrated satisfactory accuracy in monthly ET simulation for the Yellow, Yangtze, and Brahmaputra (above the Nuxia station) rivers, as gauged against ET calculations derived from the water balance method (Fig. 4). The Yellow River displayed the most robust performance, signified by the highest *Nash* value (0.77), and significantly lower *Bias* (9.0%) and *RMSE* (15.3 mm). Skill scores for both the Yangtze and Brahmaputra rivers were also within reasonable bounds. It is notable, however, that the skill scores for TPRED ET were marginally lower than those for runoff in these catchments, possibly owing to inherent errors in the water balance approach and the coarse spatial resolution of the GRACE data.

In summary, the credibility of WEB-DHM model was assessed for each headwater region by comparing the model’s outcomes with observed river discharge and ET references. The resulting high skill scores underscored the efficacy of the WEB-DHM model in accurately representing complex hydrological dynamics within cryosphere-influenced basins.

### Inter-comparison between the TPRED and other datasets

To enhance the validation of the reconstructed TPRED, it was compared with various widely recognized datasets. The assessment was done through the multi-dimensional comparison, including the monthly and annual variability, as well as spatial patterns and magnitudes across different topography and elevations.

#### Verification in runoff and ET products on the monthly scale

**Fig. 5:**
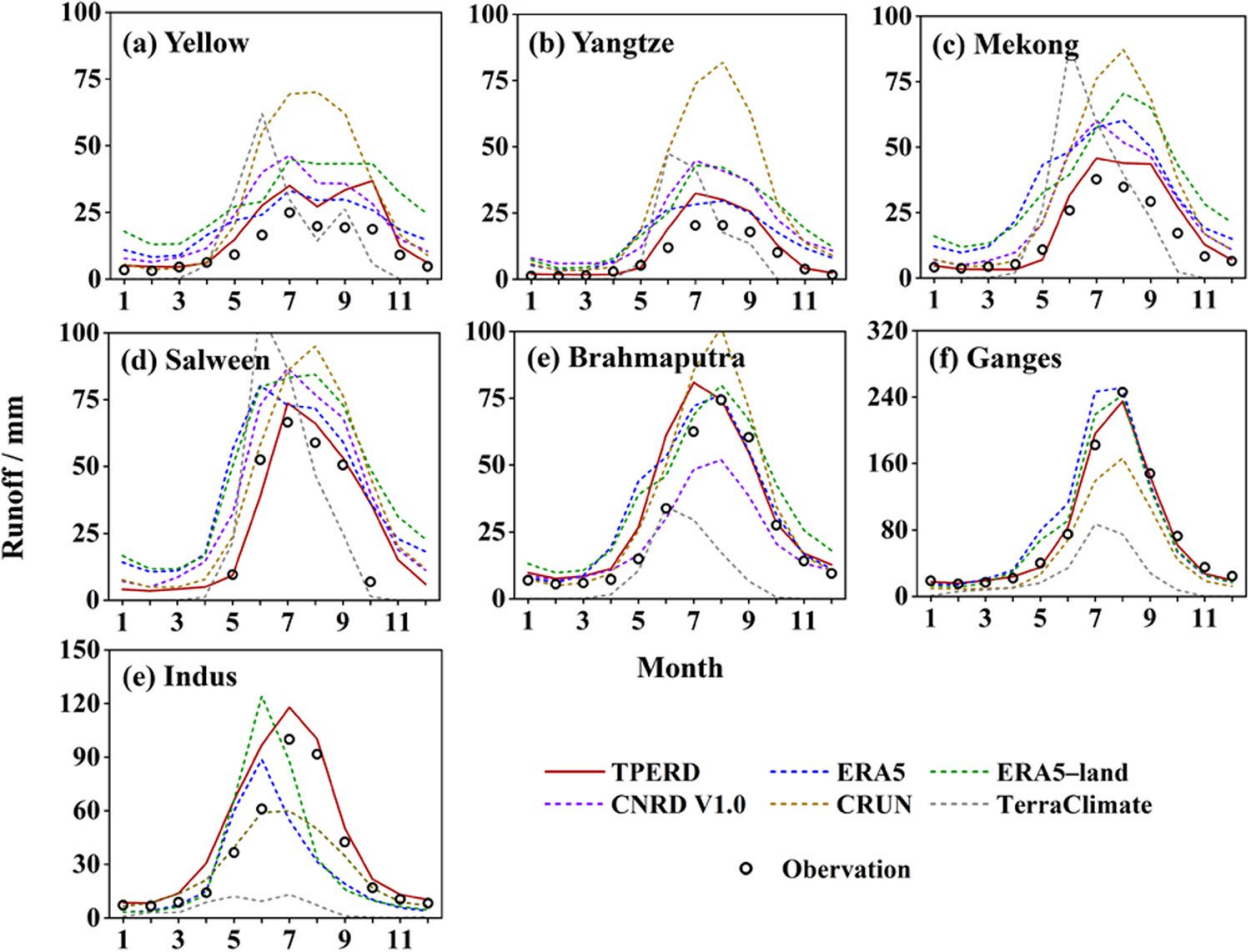
Comparison of domain-averaged monthly runoff obtained from six products with observed references averaged over the period from 1998 to 2017. The study divides the observed discharge at the outlet of each headwater by the basin area to estimate the runoff reference.

**Fig. 6:**
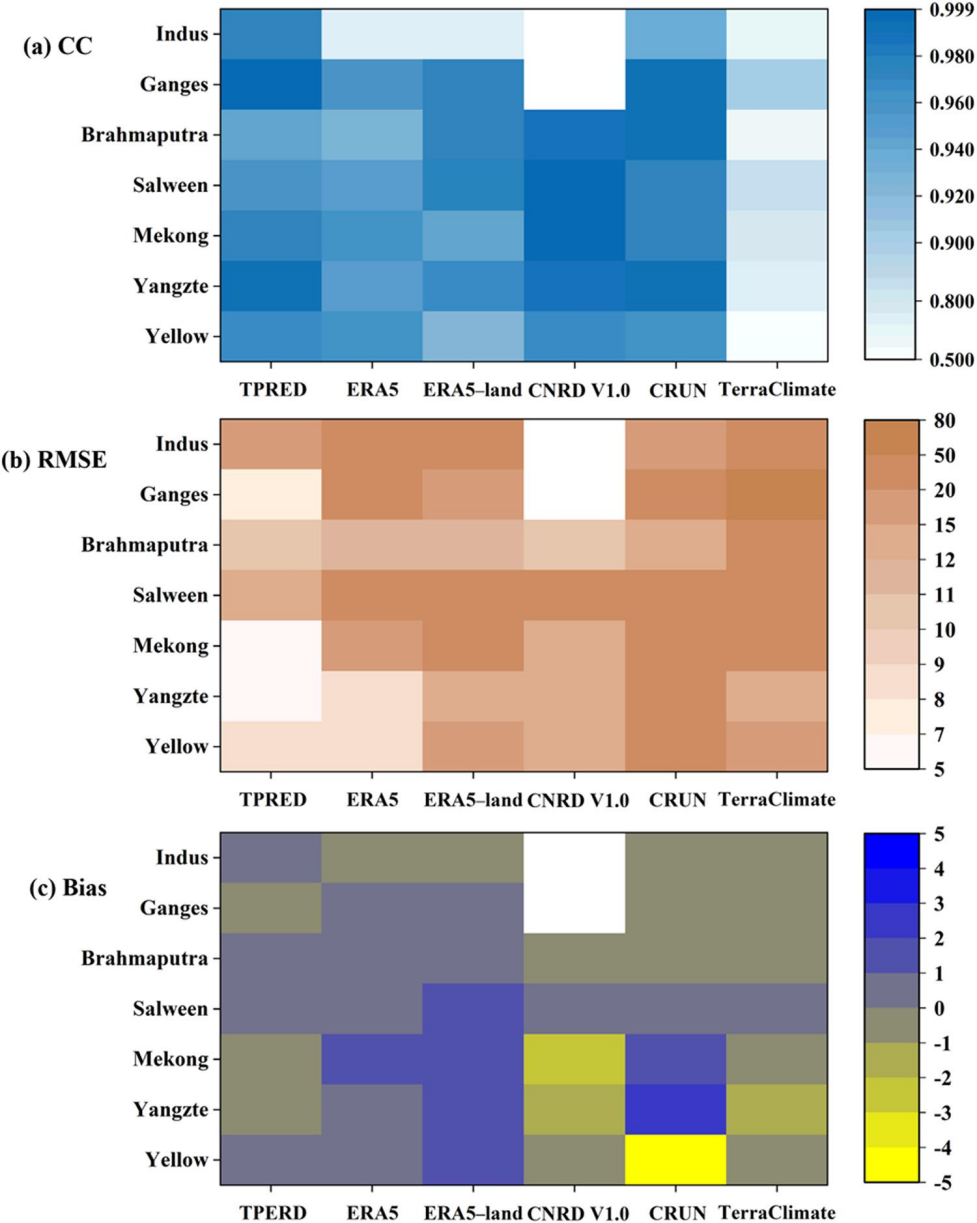
Performance metrics of each runoff product compared to observed reference. The specific indexes include (**a**) *CC*, (**b**) *RMSE*, and (**c**) *Bias*, computed based on the data presented in Fig. 5.

The monthly characteristics of six runoff datasets were examined in comparison to observational benchmarks, as illustrated in Fig. 5. The monthly runoff distribution in all datasets exhibited a consistent seasonal pattern, with values peaking during summer and reaching nadirs through winter and spring (Fig. S3). The TPRED outperformed other runoff products in capturing the temporal dynamics of high and low runoff periods, especially in recognizing “double peaks” in the Yellow headwater occurring in July and October. Additionally, the TPRED achieved high skill scores in the majority of headwater regions, supported by *CC* values exceeding 0.94. The TPRED consistently showed the lowest *RMSE* values as well as the relatively small *Bias* values when compared against other datasets (Fig. 6). In addition to TPRED, the ERA5 exhibited the second-highest agreement with observed references; CRUN significantly underestimated peak values in the Ganges and Indus headwaters while overestimating values in other basins; TerraClimate demonstrated relatively poor performance in terms of TP.

**Fig. 7:**
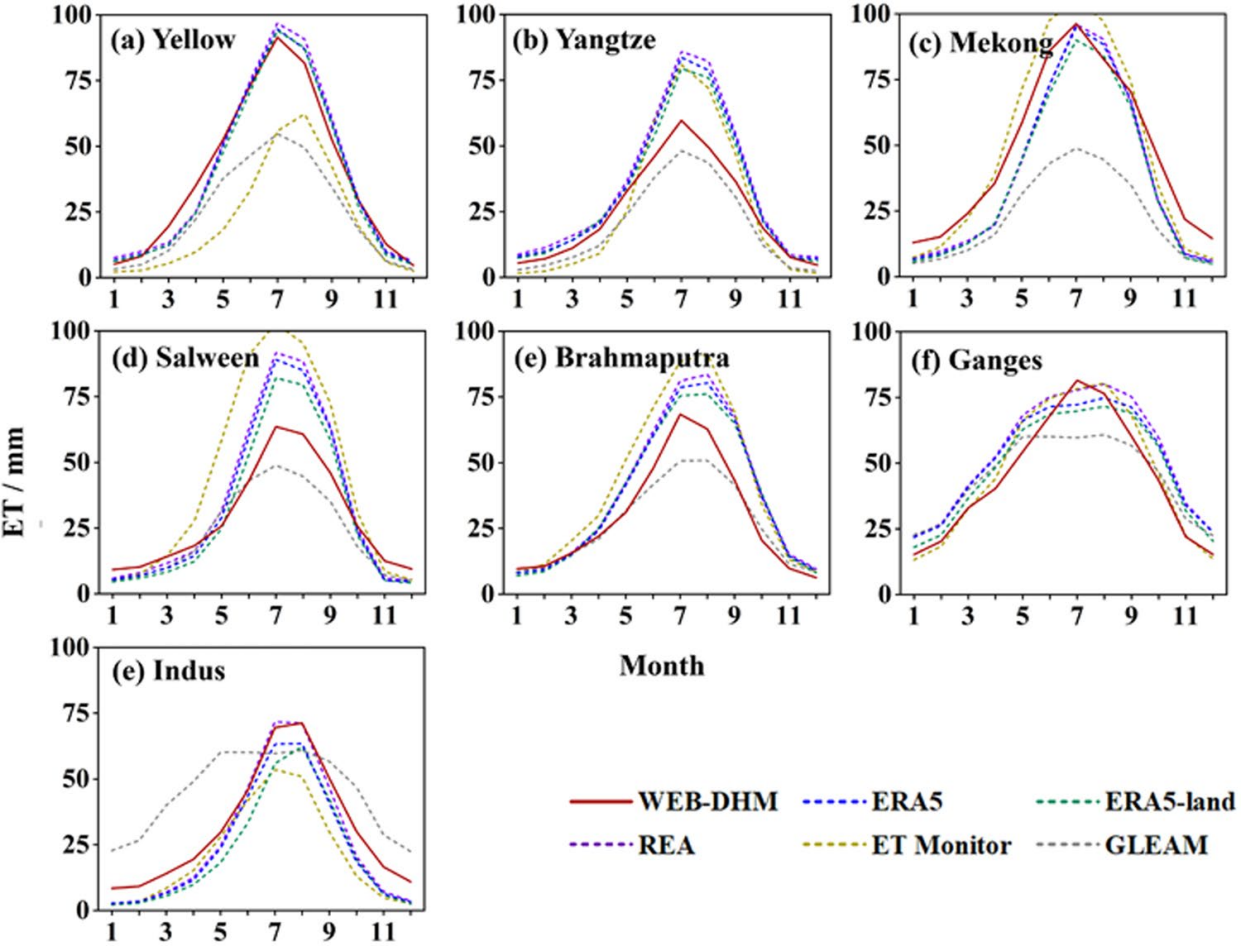
Comparison of domain-averaged monthly ET obtained from six products averaged over the period from 1998 to 2017.

**Fig. 8:**
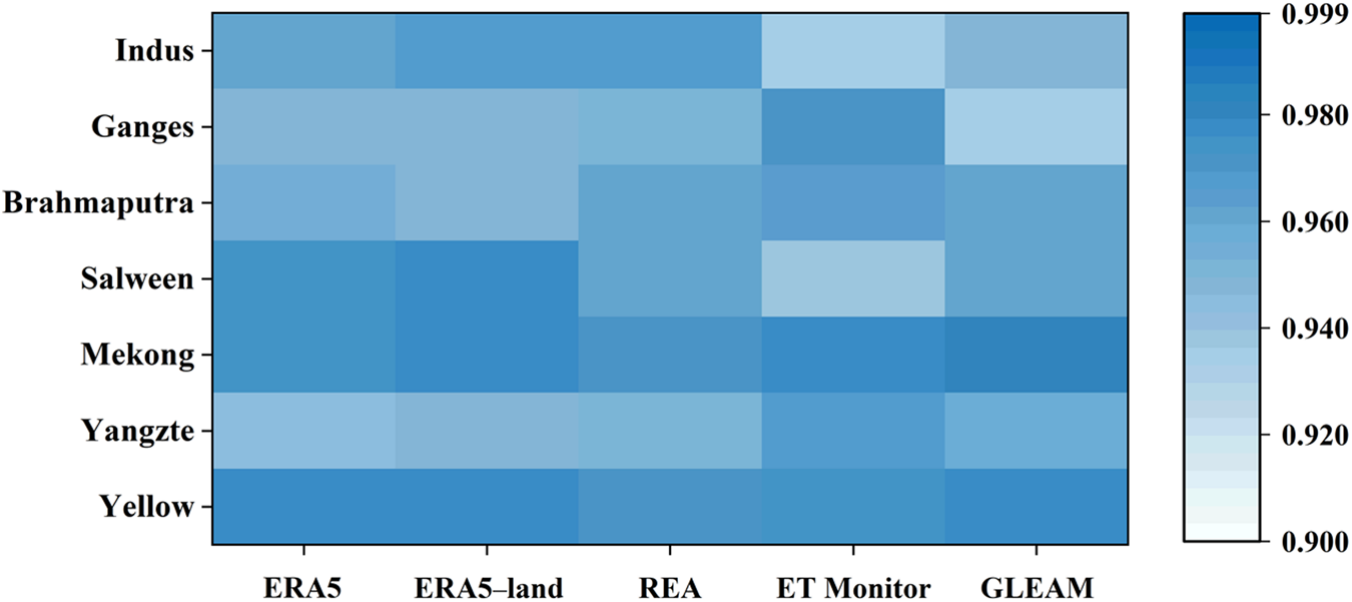
The *CC* between each ET product and TPRED on the month scale during 1998~2017.

Figure 7 illustrates the monthly distribution of ET derived from TPRED, in comparison with five alternative ET datasets. TPRED consistently captures the expected monthly ET peaks, though it exhibits slight underestimations in some rivers compared to other datasets. Notably, GLEAM ET values are generally lower than those from other products across most basins. The *CC* between TPRED and other ET products is exceptionally high on a monthly scale, with all basin values exceeding 0.935. This strong correlation indicates that TPRED’s monthly ET performance aligns well with anticipated standards (Fig. 8). The seasonal ET pattern follows the runoff pattern, with the highest ET occurring in summer and progressively lower values in autumn, spring, and winter (Fig. S4).

In brief, monthly comparisons revealed that the TPERD runoff corresponds superiorly to observed reference than other products in terms of magnitude and seasonality, and the TPRED ET estimates closely matched expected seasonal fluctuations.

#### Multi-dimensional comparison of runoff and ET products on the annual scale

**Fig. 9:**
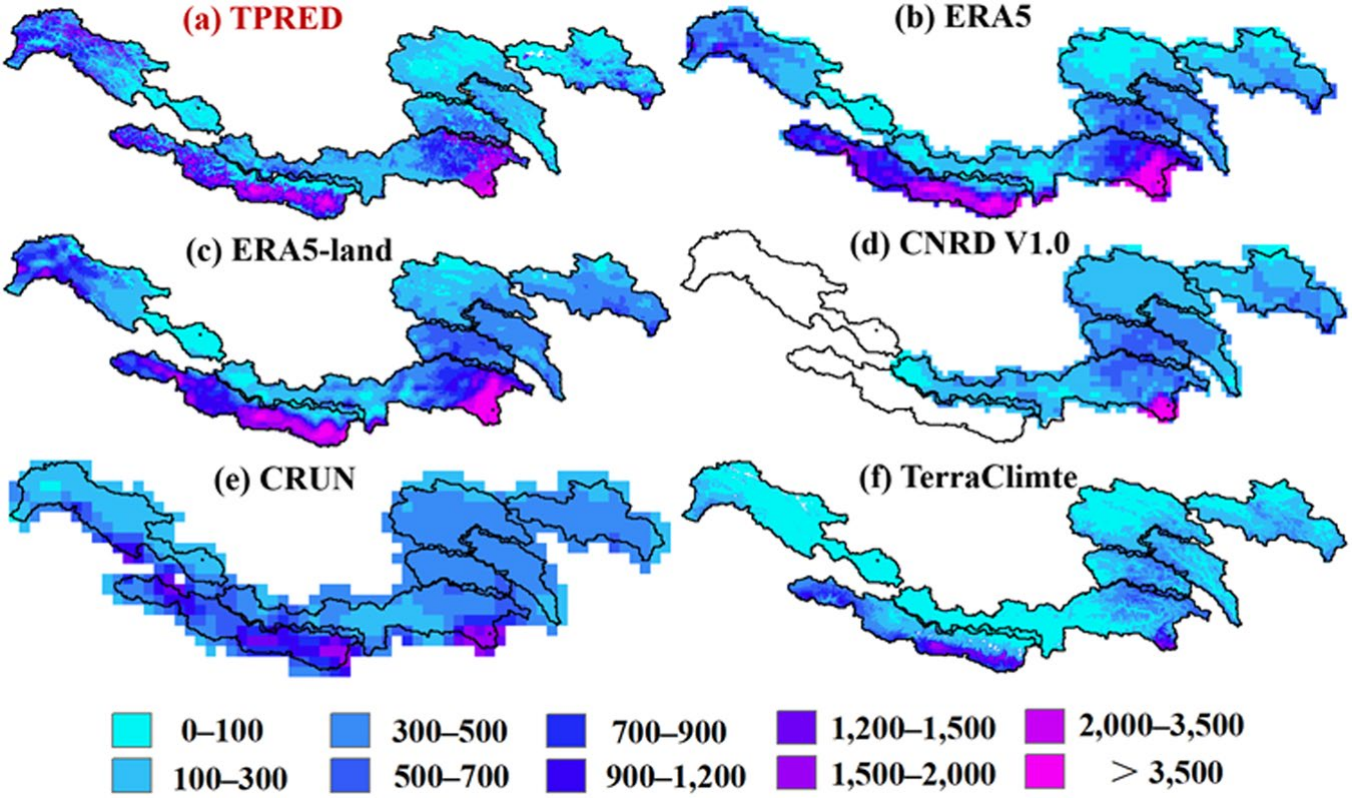
Spatial distribution of annual runoff among six products averaged over the period from 1998 to 2017 (unit: mm).

Figure 9 compares the spatial distributions of the long-term mean annual runoff as depicted by TPRED and other products. The runoff patterns portrayed by TPRED are consistent with those observed in other datasets, showing significant spatial variability. Water-rich basins, such as the Ganges, southeast Brahmaputra, and southwest Indus, contrast sharply with less affluent regions, including the Yangtze, Yellow, and Mekong basins. Variability in runoff across these basins can be largely attributed to discrepancies in rainfall distribution, as precipitation remains the primary source of runoff replenishment. This relationship is evidenced by a significant positive correlation between precipitation and runoff, excluding glacier-covered areas (Fig. S5). Furthermore, TPRED demonstrates superior capability in depicting the spatial heterogeneity of water resources across complex landscapes, especially in areas with substantial variations in elevation.

**Fig. 10:**
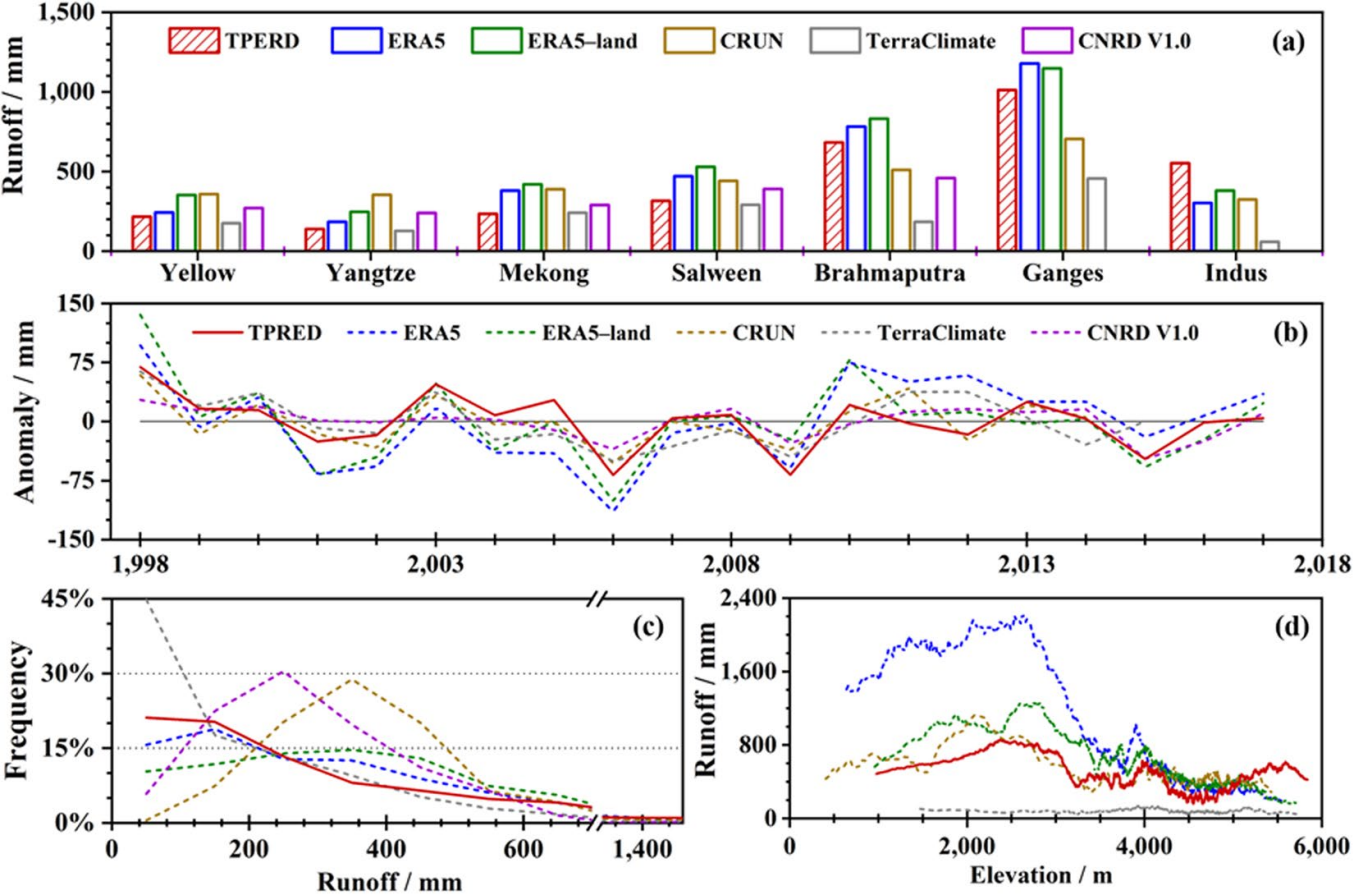
Multi-dimensional comparison on six runoff products at the annual scale across different headwater regions. (**a**) Mean annual runoff for the period 1998–2017. (**b**) Temporal variation of annual runoff anomalies, defined as the differences between annual runoff values and the multi-year average for each product. (**c**) Probability distribution. (**d**) Altitudinal distribution.

The annual average runoff from the TPRED and other datasets was compared and visualized multidimensionally, revealing concordance across basins in terms of magnitude, except for TerraClimate, which significantly underestimated runoff (Fig. 10a). TPRED runoff values varied from 140 mm (Yangtze) to 1011 mm (Ganges). Interannual anomalies showed similar trends among all datasets, with minima in 2006, 2009, and 2015, and common peaks in 2000 and 2003 (Fig. 10b). Probabilistic and altitudinal distributions demonstrated that, despite magnitude discrepancies in specific elevation ranges, the overall pattern of runoff variation with altitude was consistent across most products (Fig. 10c,d).

**Fig. 11:**
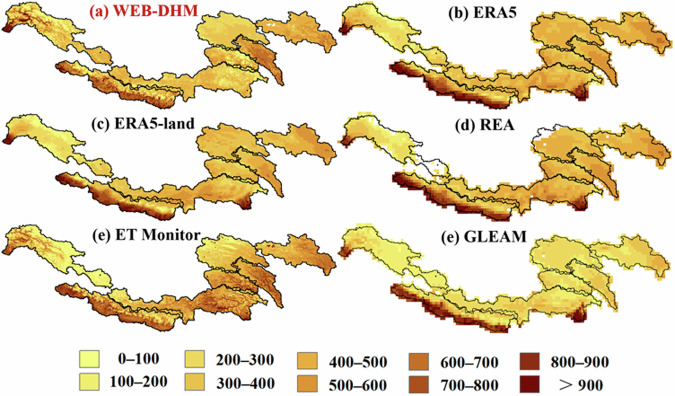
Spatial distribution of annual ET among six products averaged over the period from 1998 to 2017 (unit: mm).

**Fig. 12:**
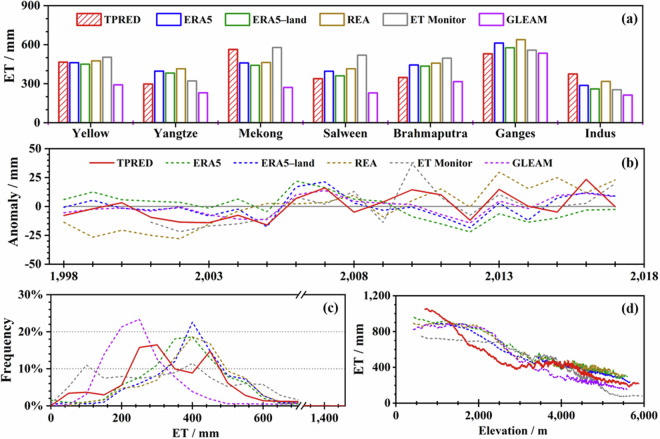
Multi-dimensional comparison on six ET products at the annual scale across different headwater regions. (**a**-**d**) Similar to Fig. 11, but for ET.

A comprehensive comparison was performed on ET datasets, as illustrated in Figs. 11 and 12. The southern Ganges and western Indus headwaters exhibited the highest ET values, whereas the Yangtze showed the lowest annual average ET. The Yellow and Mekong presented higher ET values than expected, given their respective precipitation and runoff levels. In the contexts of spatial distribution, probabilistic behavior, and altitudinal gradients, the ERA5 dataset displayed the greatest congruence with TPRED. The GLEAM consistently underestimated the magnitude of ET and failed to accurately capture interannual variability. The ERA5 dataset demonstrated inverse behavior concerning interannual fluctuations compared to most other datasets. The ET Monitor’s performance was closely aligned with the REA, although it showed a slight overestimation. Generally, ET decreased with elevation, with TPRED indicating slight fluctuations between 3000 and 4000 meters.

Collectively, the annual values of TPERD generated by the rigorously validated WEB-DHM model showed commendable alignment with other established products across multiple dimensions, including watershed-scale magnitudes, temporal deviations, and elevational gradients. TPERD, along with other products, demonstrated similar spatial variability in runoff and ET distributions. The superior spatial resolution of TPERD enabled a more detailed delineation of spatial heterogeneity in runoff and ET across topographically complex terrains compared to coarser-grained datasets.

## Supplementary information

Revised Clean Supplement

## Data Availability

The cryosphere-hydrology model (WEB-DHM) is described in detail in our regional study^59–71,80^. Detailed R codes for comparing and reanalyzing numerous runoff and ET products in this study is available in the Supplement “Part 3”.
