# Supplementary material for: Tibetan Plateau Runoff and Evapotranspiration Dataset by an observation-constrained cryosphere-hydrology model
Source: Sci Data. 2024 Jul 13;11:773. doi: 10.1038/s41597-024-03623-3 (PMC11246465; doi:10.1038/s41597-024-03623-3)
Supplement: Supplementary file 1 — Revised Clean Supplement [file 41597_2024_3623_MOESM1_ESM.docx]

**Supplement for**

**Tibetan Plateau Runoff and Evapotranspiration Dataset by an observation‒constrained cryosphere‒hydrology model**

**Catalogue**

[Part 1: Details of datasets used in this study 2](#_Toc168080012)

[Part 1.1: Datasets used in model input and evaluation 2](#_Toc168080013)

[Part 1.2: Limitations of the state-of-the-art runoff and ET products in the TP 4](#_Toc168080014)

[Part 2: Figures 8](#_Toc168080015)

[Part 3: R Codes 13](#_Toc168080016)

[Part 3.1: Convert the raw product in “.tif” or “.nc” format into monthly, seasonal and annual data in “.tif” format, respectively. 13](#_Toc168080017)

[Part 3.2: Compute time series values for months and years. 16](#_Toc168080018)

[Part 3.3: Calculate the altitude gradient of the product. 18](#_Toc168080019)

[Part 3.4: Application example of hydrometeorology. 20](#_Toc168080020)

[Part 3.5: Application example of carbon transport. 23](#_Toc168080021)

[Part 3.6: Spatial correlation analysis of runoff and precipitation. 25](#_Toc168080022)

[Part 4: References 27](#_Toc168080023)

# Part 1: Details of datasets used in this study

## Part 1.1: Datasets used in model input and evaluation

Prior to model construction, it is crucial to collate both basic and driving input datasets. The basic datasets include: (1) Digital Elevation Model (DEM) data acquired from the Shuttle Radar Topography Mission (SRTM), available at http://srtm.csi.cgiar.org/ with a 90 m resolution; (2) Land use data sourced from the United States Geological Survey (USGS) database, accessible at http://edc2.usgs.gov/glcc/glcc.php, comprising 11 land use classifications; (3) Soil type data derived from the global digital soil dataset published by the Food and Agriculture Organization (FAO) of the United Nations, retrievable at http://www.fao.org/geonetwork/srv/en/main.home; (4) Soil organic carbon data, segmented into seven soil depth levels, available from the International Soil Reference and Information Center (ISRIC) at https://soilgrids.org/; (5) Glacier data, which is specific to basin characteristics, includes the Second Glacier Inventory of China with data on glacier changes on the Tibetan Plateau (TP) based on Landsat satellite images^1–4^, and data from the International Center for Integrated Mountain Development (ICIMOD)^5^, which is generated using a semi-automated classification method employing various sources including Landsat and Google Earth imagery.

Forcing datasets encompass both vegetation and climate. Vegetation dynamics were obtained using the Global Land Surface Satellite (GLASS) products^6^, which provide Leaf Area Index (LAI) and Fraction of Photosynthetically Active Radiation (FPAR) data. Meteorological forcings consisted of variables such as precipitation, air temperature, relative humidity, atmospheric pressure, and wind speed, along with both shortwave and longwave radiation. Various meteorological products were utilized, including the China Meteorological Administration (CMA), China Meteorological Forcing Dataset (CMFD)^7^, Tibetan Plateau Meteorological Forcing Dataset (TPFMD)^8^, ERA5^9^, ERA5-Land^10^, or Global Land Data Assimilation System (GLDAS)^11^. To enhance the precision of the model outcomes, our team has developed an innovative suite of precipitation datasets at various river basins (e.g., upper Brahmaputra^12^), by integrating various observational, satellite, and reanalysis datasets. Additionally, the integrated precipitation datasets employed in each river basin will undergo rigorous assessment against limited ground-based observations to determine the most appropriate datasets.

Following the acquisition of the aforementioned datasets, *in situ* daily discharge observations at the outlets of each headwater (as depicted in Fig.1a) were employed to calibrate the WEB-DHM model. Notably, data from the Nuxia station (Brahmaputra River) and the Karnali station (Ganges tributary) were obtained from gauge measurements conducted as part of the TP-River project, while observed data from other hydrologic stations were sourced from respective national authorities, including the Ministry of Water Resources of China (http://www.mwr.gov.cn/english/) and the Water and Power Development Authority of Pakistan (WAPDA)^13^. *In situ* observations of soil temperature/moisture, particularly from the headwaters of the Yellow River, were also compiled to assess the model’s accuracy^14,15^. Additionally, remote sensing products were utilized to validate the model outputs. To evaluate the model’s proficiency in simulating land surface temperature (LST), data from the Moderate Resolution Imaging Spectroradiometer (MODIS) with a spatial resolution of 1 km and a temporal resolution of 8 days were used^16^. Furthermore, the validation of snow depth simulations relied on daily data from both the SSM/I product from satellite passive microwave remote sensing data (https://data.tpdc.ac.cn/zh-hans/data/47acf141-2c86-4e1f-8f68-036ae57d268e) and the M*D10A1GL06 product, which integrates daily Terra and Aqua MODIS snow observations with the Randolph Glacier Inventory 6.0^17^.

## Part 1.2: Limitations of the state-of-the-art runoff and ET products in the TP

Although there has been significant progress in the development of global or regional hydrological products, acquiring reliable runoff and ET datasets across the TP remains challenging. This issue is primarily due to the lack of observational data essential for product calibration and validation. Additionally, conventional methodologies and models often fail to adequately consider the cryosphere-hydrological processes related to glaciers, snow, and permafrost. Furthermore, the coarse spatial resolution of many products fails to capture the fine-scale spatial heterogeneity in high-mountain basins. These limitations are discussed in detail below.

The reliability of existing runoff and ET datasets across the TP is compromised by insufficient observational data^18^. For instance, the widely recognized observation-based gridded runoff dataset CRUN underperforms in the TP due to sparse observational inputs^19^. Other runoff products such as the fifth generation of European Reanalysis (ERA5) ^9^, its new land component (ERA5-Land)^10^, the China Natural Runoff Dataset version 1.0 (CNRD v1.0)^20^, and river discharge products from the Global Flood Awareness System (GLoFAS)^21^, the adjusted Japanese 55-year reanalysis (JRA-55)^22^, the global reconstruction of naturalized river flows (GRNRF)^23^, and its derived reach-level flood reanalysis (GRFR)^24^, also suffer from inadequate observational data for model constraint or output validation over the TP. Similarly, ET products across the TP face challenges due to insufficient observational support for calibration and validation^25,26^. Recognized ET models such as the Global Land Evaporation Amsterdam Model (GLEAM) and ET Monitor^27^ are hindered by a lack of flux tower data on the TP. Other datasets like SiTHv2^28^ and the PEW^29^ model also lack verification against river basin water balances, particularly in plateau-origin basins. Moreover, combined global ET products using the reliability ensemble averaging (REA) method across datasets such as ERA5, GLDAS2, and MERRA-2 show significant inconsistencies in High Mountain Asia due to the lack of observations and large discrepancies between products^30^. Both the CAMELE^31^ and HG-Land ET^32^ datasets, which integrate multiple ET datasets, have only one flux tower verification point in the TP.

Furthermore, many current gridded runoff and ET products do not incorporate fundamental cryosphere hydrophysical processes such as those involving snow, glaciers, and frozen soil. For example, GRUN runoff creation uses machine learning to form regressions between *in situ* discharge observations and meteorological variables, yet its performance is weakened in data-scarce areas and excludes cryosphere considerations^19^. The CNRD v1.0, derived from the VIC model, demonstrates optimal regionalization for model parameters in pseudo-/test-ungauged catchments but lacks physical parameterization schemes for essential cryosphere hydrological processes^20^. Channel discharge datasets from GloFAS^21^, JRA-55^22^, GRNRF^23^, and GRFR^24^ also lack nuanced treatment for cryosphere catchments despite employing advanced river routing modeling techniques. For ET products, common approaches involve integrating multiple existing products using mathematical and statistical methods, but these approaches inherently lack detailed explanations for underlying land surface hydrology and energy processes^30–32^. ET models such as GLEAM^33^, ET Monitor^27^, SiTHv2^28^, and PEW^29^ can offer insights into surface energy processes but still omit hydrothermal interactions prevalent in permafrost regions.

The HTESSEL-based ERA5 products provide long-term, spatio-temporally continuous runoff and ET data with improved snowpack parameterization and bare soil evaporation. However, they lack independent glacier treatment, using a fixed snow mass for grid points with glacial coverage^9^. ERA5-Land partially accounts for snow, glacier, and frozen soil processes. Although frequently applied in global-scale hydrometeorological studies^34–36^, its effectiveness in high-altitude mountainous areas is diminished, as validated by multiple studies^10,37–39^. Advances in accurately representing 2-meter temperature and snow levels, datasets derived from ERA5-Land, such as GloFAS, poorly characterize peak river flows in regions predominantly influenced by snowmelt, especially at high latitudes^40^. This can be attributed to its simplistic approach to cryosphere hydrophysical processes, including a single-layer snow model, a glacier mask derived from snow cover, and updated parameterization for soil thermal conductivity^10,40^. In contrast, the WEB-DHM model’s enthalpy-based three-layer snow and permafrost modules, and energy-based glacier modules, offer a more sophisticated representation. Although ERA5 has limitations in portraying cryosphere hydrophysical mechanisms compared to ERA5-Land, it demonstrates superior accuracy in estimating snowpack, river flow, and ET in high mountains due to the lack of land data assimilation in ERA5-Land^10,39^.

The relatively coarse spatial resolution of existing products further limits the accurate portrayal of the finer-scale spatial heterogeneity in TP water resources. The constraints imposed by the spatial scale of hydrological modeling limit the precise simulation of cryosphere hydrophysical processes, including the dynamics of debris-covered glaciers, avalanches, the impact of wind on snow transport, and the presence of glacial lakes^41^. High-resolution modeling, with spatial resolution finer than 10 km, offers significant advantages by enabling a more detailed exploration of complex processes such as gravity-driven snow redistribution, topographic shading effects, supraglacial cliffs and ponds, minor glacier elevation fluctuations, and avalanche mechanisms^18,42–44^. Employing this high-resolution approach significantly improves the accuracy of hydrological simulations over methodologies that use lower resolutions. Hence, the access to high-resolution datasets is crucial for capturing fine-scale hydrological processes within cryospheric domains. However, there is a noticeable gap in the availability of high-resolution datasets across the TP compared to other regions.

Table 3 presents the runoff and ET datasets utilized in this research for cross-comparison validation. The datasets listed in Table 3 are predominantly global, except for CNRD V1.0, which is specific to China. Direct comparisons between global or national datasets and the regional TPRED are generally not considered suitable. However, due to a distinct lack of runoff and ET datasets specifically for the TP, no appropriate alternatives exist for an exhaustive parallel comparison with TPRED, necessitating the use of relevant segments from global datasets. Products like CNRD V1.0 and CRUN benefit from a modest level of observation for validation on the TP but do not sufficiently address hydrological processes involving snow, glaciers, and permafrost. Conversely, datasets such as ERA5, ERA5-Land, and TerraClimate, although unvalidated against direct observations for the TP, do consider ice and snow processes to some extent. Furthermore, the validation of existing ET products using flux tower data is notably lacking in the TP, and many associated research methodologies inadequately address the complex surface processes characteristic of this region compared to hydrological and land surface.

# Part 2: Figures


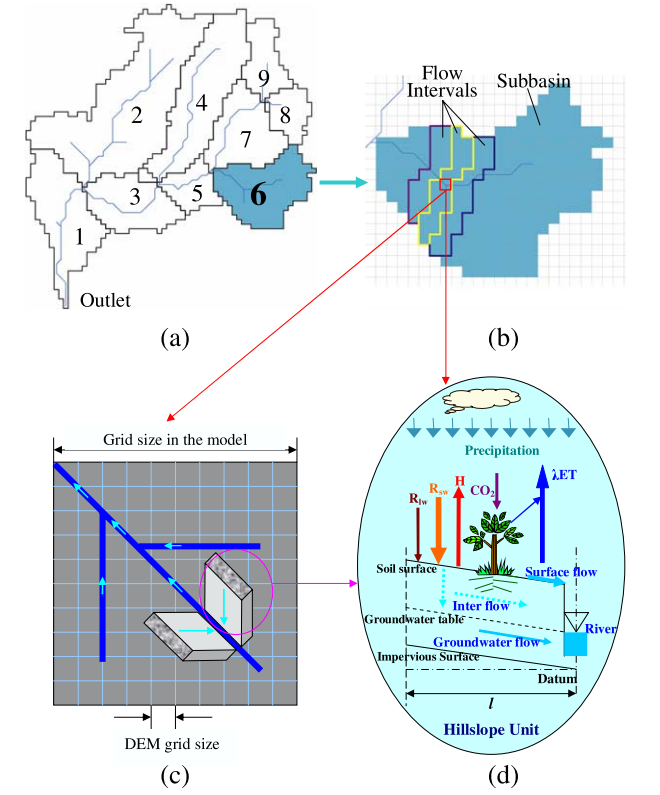


**Figure S1. Overall structure of the WEB-DHM**^45^**.** (a) division from a basin to subbasins, (b) subdivision from a subbasin to flow intervals comprising several model grids, (c) discretization from a model grid to a number of geometrically symmetrical hillslopes, and (d) process descriptions of water moisture transfer from the atmosphere to river. Here the SiB2 is used to describe the transfer of the turbulent fluxes (energy, water, and CO_2_ fluxes) between the atmosphere and land surface for each model grid, where *R_sw_* and *R_lw_* are downward solar radiation and longwave radiation, *H* is the sensible heat flux, and *λ* is the latent heat of vaporization. The GBHM simulates both surface and subsurface runoff using grid-hillslope discretization, and then simulates flow routing in the river network.


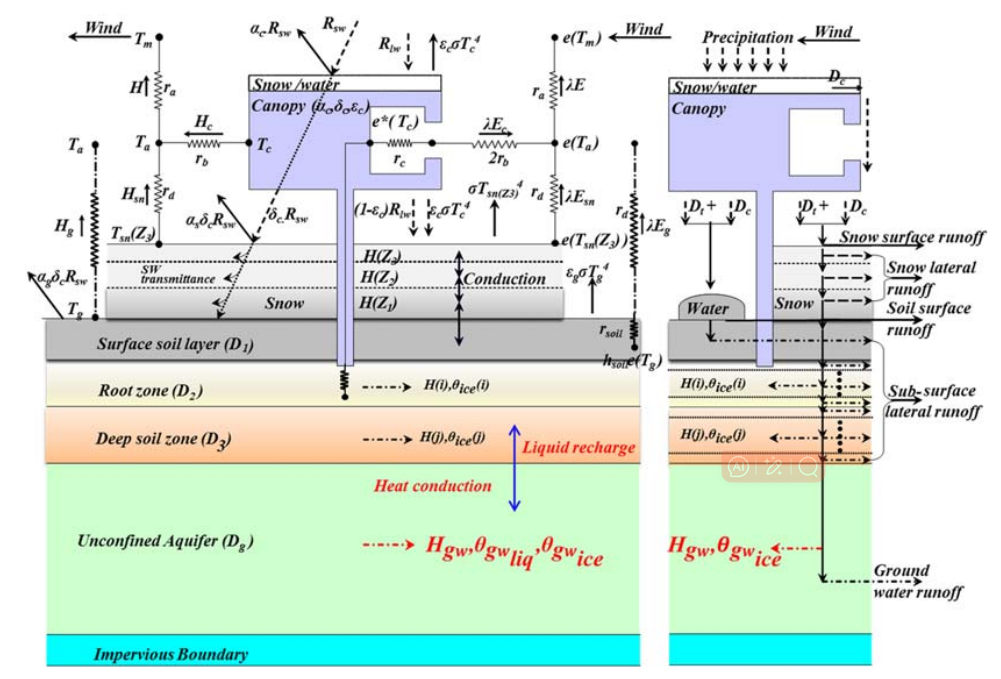


**Figure S2. Structure of the WEB-DHM model**^15,46^**.** This model considers the water phase change in the unconfined aquifer and its exchanges of water and heat with the upper soil layers, with enthalpy adopted as a prognostic variable instead of soil temperature in the energy balance equation to avoid instability when calculating water phase changes. *T* is temperature, *e(T)* is vapor pressure at *T*, *R_sw_* and *R_lw_* are downward shortwave and longwave radiation, *H* and *λE* are sensible and latent heats, and *ε*, *δ* and *α* are emissivity, transmittance, and reflectance, respectively. *H(i)* and *H(j)* in the right part are the soil enthalpy used in the frozen soil module, while *θ_ice(i)_* and *θ_ice(j)_* represent the soil ice content in the frozen soil module. Subscript *c* refers to canopy, *g* to soil surface, *sn* to snow surface, and *m* to the reference height^46^. *H_gw_* is the enthalpy, and *θ_gwliq_* and *θ_gwice_* represent the liquid water content and ice content in the unconfined aquifer, respectively^15^.


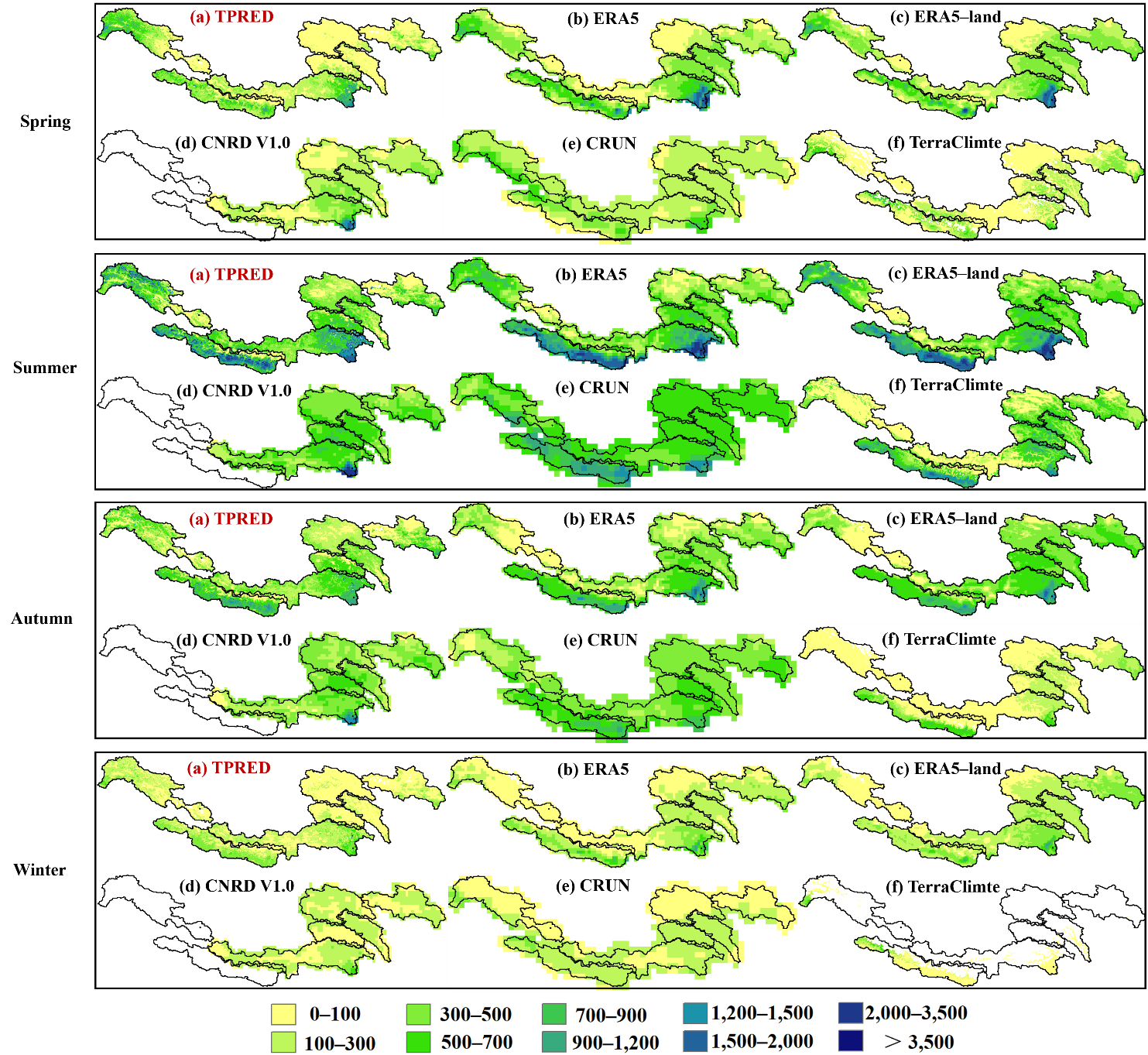


**Figure S3. Spatial distribution of seasonal runoff among six products averaged over the period from 1998 to 2017 (unit: mm).**

**
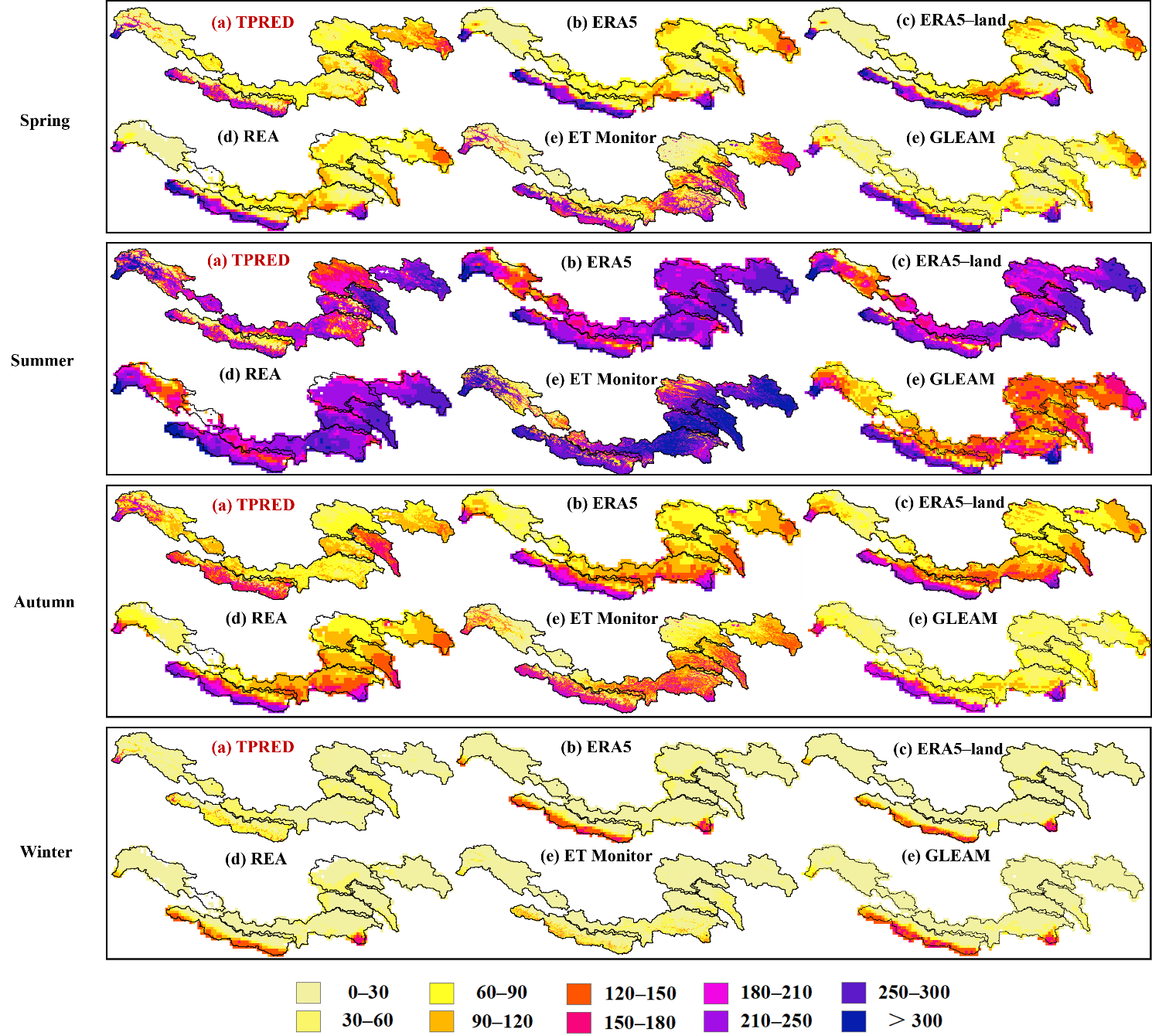
**

**Figure S4. Spatial distribution of seasonal ET among six products averaged over the period from 1998 to 2017 (unit: mm).**


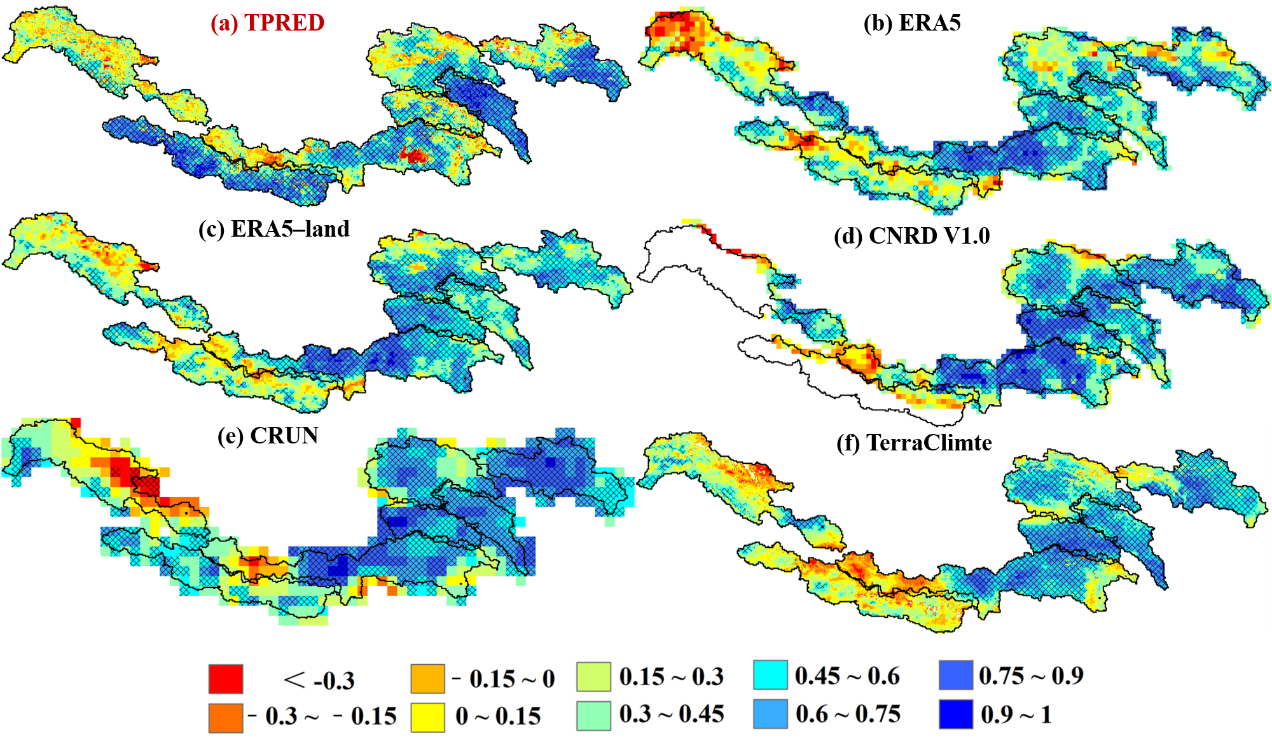


**Figure S5. Spatial patterns of the correlation coefficient (CC) between annual runoff and precipitation (TPFMD product^8^) across the seven headwater regions of TP for the period 1998‒2017. The shadow denotes that the shift in trend has undergone statistical significance testing.**

# Part 3: R Codes

In this study, a meticulous comparison was conducted between the TPRED gridded runoff and ET product and major established gridded datasets. These comparisons involved runoff products from ERA5, ERA5‒Land, CNRD V1.0, CRUN, and TerraClimate, as well as ET products from ERA5, ERA5‒Land, REA, ET Monitor, and GLEAM.

The subsequent section takes TPRED and ERA5-land products as the example to provide the detailed **R codes** of the data processing, specifically focused on the headwaters of Yellow river.

## Part 3.1: Convert the raw product in “.tif” or “.nc” format into monthly, seasonal and annual data in “.tif” format, respectively.

This section exemplifies the processing of ERA5‒land runoff.

library(data.table)

library(raster)

library(ncdf4)

library(lattice)

library(sp)

library(rgdal)

library(gstat)

library(sf)

**### *Read the raw ERA5‒land runoff.***

setwd("‘home\\00-ERA5-land-runoff-process")

input_nc="ERA5-land_runoff_0.1_monthly_average_1998_2017.nc"

varname="ro"

raster_runoff=stack(input_nc, varname="ro")

raster_runoff

time_numbers <- 1:nlayers(raster_runoff)

length(time_numbers)

monthdays <- read.csv("monthdays.csv")

date <- as.matrix(monthdays)

**### *Reading watershed boundaries***

bound0<-raster("C:/Users/fanxi/Desktop/00-ERA5-land-runoff-process/bound_WHB_tif/Yellow.tif")

WGS84<- CRS("+proj=longlat +datum=WGS84 +no_defs +ellps=WGS84 +towgs84=0,0,0")

bound<- projectRaster(bound0,crs=WGS84)

projection(raster_runoff) = WGS84

raster_runoff

raster_runoff_7 <- raster_runoff[[7]]

raster_runoff_7

bound_resample <- resample(bound, raster_runoff_7, method = "bilinear")

bound_resample

**### *Export monthly data.***

setwd("‘home\\00-ERA5-land-runoff-process\\0_Yellow_month")

for(i in 1:length(time_numbers)){

selection<-date[i,]

name<-paste(selection[1], "-", selection[2], " ","runoff"," ","-Yellow",sep="")

mask_raster_runoff <- mask(raster_runoff[[i]], bound_resample)

mask_raster_runoff_value <- as.data.frame(mask_raster_runoff)

names(mask_raster_runoff_value) <- "runoff"

monthday <- selection[3]

mask_raster_runoff_value$runoff <- mask_raster_runoff_value$runoff*monthday*1000

values(mask_raster_runoff) <- mask_raster_runoff_value$runoff

writeRaster(mask_raster_runoff, name, format = 'GTiff', overwrite = TRUE)

}

**### *Transfrom the monthly data into seasonal data.***

year<-rep(1998:2017)

for (i in 1:length(year)){

setwd("‘home\\00-ERA5-land-runoff-process\\Yellow_month")

year_name = year[i]

name01 <- paste(year_name, "-1-Yellow-clip.tif",sep="")

name02 <- paste(year_name, "-2-Yellow-clip.tif",sep="")

name03 <- paste(year_name, "-3-Yellow-clip.tif",sep="")

name04 <- paste(year_name, "-4-Yellow-clip.tif",sep="")

name05 <- paste(year_name, "-5-Yellow-clip.tif",sep="")

name06 <- paste(year_name, "-6-Yellow-clip.tif",sep="")

name07 <- paste(year_name, "-7-Yellow-clip.tif",sep="")

name08 <- paste(year_name, "-8-Yellow-clip.tif",sep="")

name09 <- paste(year_name, "-9-Yellow-clip.tif",sep="")

name10 <- paste(year_name, "-10-Yellow-clip.tif",sep="")

name11 <- paste(year_name, "-11-Yellow-clip.tif",sep="")

name12 <- paste(year_name, "-12-Yellow-clip.tif",sep="")

raster_spring <- stack(list(name03, name04, name05))

raster_summer <- stack(list(name06, name07, name08))

raster_autumn <- stack(list(name09, name10, name11))

raster_winter <- stack(list(name12, name01, name02))

raster_spring_sum <- sum(raster_spring, na.rm=TRUE)

raster_summer_sum <- sum(raster_summer, na.rm=TRUE)

raster_autumn_sum <- sum(raster_autumn, na.rm=TRUE)

raster_winter_sum <- sum(raster_winter, na.rm=TRUE)

name_spring <- paste(year_name, "-spring-", "Q-", "Yellow", sep="")

name_summer <- paste(year_name, "-summer-", "Q-", "Yellow", sep="")

name_autumn <- paste(year_name, "-autumn-", "Q-", "Yellow", sep="")

name_winter <- paste(year_name, "-winter-", "Q-", "Yellow", sep="")

setwd("‘home\\00-ERA5-land-runoff-process\\seasons\\spring")

writeRaster(raster_spring_sum, name_spring, format = 'GTiff', overwrite = TRUE)

setwd("‘home\\00-ERA5-land-runoff-process\\seasons\\summer")

writeRaster(raster_summer_sum, name_summer, format = 'GTiff', overwrite = TRUE)

setwd("‘home\\00-ERA5-land-runoff-process\\seasons\\autumn")

writeRaster(raster_autumn_sum, name_autumn, format = 'GTiff', overwrite = TRUE)

setwd("‘home\\00-ERA5-land-runoff-process\\seasons\\winter")

writeRaster(raster_winter_sum, name_winter, format = 'GTiff', overwrite = TRUE)

}

**### *Transfrom the monthly data into annual data.***

year<-rep(1998:2018)

for (i in 1:length(year)){

year_name = year[i]

setwd("‘home\\00-ERA5-land-runoff-process\\0_Yellow_month")

name_select <- paste(year_name, ".", "*", ".", "tif", "$", sep="")

raster_runoff_year <- stack(list.files(pattern = name_select))

raster_runoff_year_sum <- sum(raster_runoff_year, na.rm=TRUE)

name <- paste(year_name, "-Yellow", ".tif",sep="")

setwd("‘home\\00-ERA5-land-runoff-process\\0_Yellow_year")

writeRaster(raster_runoff_year_sum, name, format = 'GTiff', overwrite = TRUE)

}

**### *Calculate and output multi-year mean data.***

setwd("‘home\\00-ERA5-land-runoff-process\\0_Yellow_year")

name_select <- paste(".", "*", ".", "tif",sep="")

raster_runoff_year <- stack(list.files(pattern = name_select))

raster_runoff_year_mean <- mean(raster_runoff_year, na.rm=TRUE)

raster_value <- as.data.frame(raster_runoff_year_mean, xy = TRUE)

head(raster_value)

colnames(raster_value) <- c("lon", "lat", "data")

library(data.table)

raster_value <- as.data.table(raster_value)

raster_value[data == 0, data:= NA]

values(raster_runoff_year_mean) <- c(raster_value$data)

raster_runoff_year_mean

name <- paste("000_Yellow-ERA5-land-runoff-", "1998-2017", "-", "mean", sep="")

setwd("‘home\\00-ERA5-land-runoff-process")

writeRaster(raster_runoff_year_mean, name, format = 'GTiff', overwrite = TRUE)

## Part 3.2: Compute time series values for months and years.

This section exemplifies the processing of TPRED runoff product only focused on the headwaters of Yellow river.

**### *Compute time series values for months.***

year<-rep(1998:2017,each=12)

month<-rep(01:12,20)

date<-cbind(year,month)

class(date)

head(date)

tail(date)

dim(date)

data = as.data.frame(matrix(nrow = length(year), ncol=3))

colnames(data) <- c("year", "month", "data")

setwd("‘home\\00-ERA5-land-runoff-process\\Yellow_month")

rawpath <- "‘home\\00-ERA5-land-runoff-process\\Yellow_month"

prefiles <- list.files(path = rawpath, full.names = T,pattern = "^.*.tif")

for (i in 1:length(year)){

selection<-date[i,]

name <- paste(selection[1], "-", selection[2], "-Yellow.tif",sep="")

AAA_i <- raster(name)

AAA_i_value <- as.data.frame(AAA_i, xy = TRUE)

colnames(AAA_i_value) <- c("lon", "lat", "AAA")

AAA_i_value <- as.data.table(AAA_i_value)

AAA_i_mean <- mean(AAA_i_value$AAA, na.rm=TRUE)

data[i,1] = selection[1]

data[i,2] = selection[2]

data[i,3] = AAA_i_mean

}

setwd("‘home\\00-ERA5-land-runoff-process")

write.csv(data, "001-Yellow-runoff-1998-2017_month.csv", row.names=FALSE)

**### *Compute time series values for years.***

setwd("‘home\\00-ERA5-land-runoff-process\\Yellow_year")

year<-rep(1998:2017)

data = as.data.frame(matrix(nrow = length(year), ncol=2))

colnames(data) <- c("year", "data")

rawpath <- "‘home\\00-ERA5-land-runoff-process\\Yellow_year"

prefiles <- list.files(path = rawpath, full.names = T,pattern = "^.*.tif")

for (i in 1:length(year)){

name <- prefiles[i]

AAA_i <- raster(name)

AAA_i_value <- as.data.frame(AAA_i, xy = TRUE)

colnames(AAA_i_value) <- c("lon", "lat", "AAA")

AAA_i_value <- as.data.table(AAA_i_value)

AAA_i_value[AAA == 0, AAA := NA]

AAA_i_mean <- mean(AAA_i_value$AAA, na.rm=TRUE)

data[i,1] = year[i]

data[i,2] = AAA_i_mean

}

setwd("‘home\\00-ERA5-land-runoff-process")

write.csv(data, "001-Yellow-runoff-1998-2017_year.csv", row.names=FALSE)

## Part 3.3: Calculate the altitude gradient of the product.

setwd("‘home\\001-calculate-DEM_altitude-and-TPRED_runoff")

bound<-raster("Yellow.tif")

raster_choose <- raster("000-TPRED-runoff-year-1998~2017-mean.tif")

bound_resample <- resample(bound, raster_choose, method = "bilinear")

bound_resample_crop <- crop(bound_resample, bound)

bound <- bound_resample_crop

runoff_raster_choose <- raster("000-TPRED-runoff-year-1998~2017-mean.tif")

runoff_raster_choose_crop <- crop(raster_choose, bound)

runoff_raster_choose_crop_mask <- mask(runoff_raster_choose_crop, bound)

plot(runoff_raster_choose_crop_mask)

name <- paste("000-TPRED-runoff-year-1998~2017-mean", ".tif",sep="")

writeRaster(runoff_raster_choose_crop_mask, name, format = 'GTiff', overwrite = TRUE)

runoff_raster_value <- as.data.frame(runoff_raster_choose_crop_mask, xy = TRUE)

colnames(runoff_raster_value) <- c("lon", "lat", "runoff")

write.table(runoff_raster_value, “Yellow-runoff-01.csv", row.names=FALSE, col.names=TRUE, sep=" ")

raster_choose_dem <- raster("000-Gobal-DEM.tif")

raster_choose_dem_crop <- crop(raster_choose_dem, bound)

raster_choose_dem_crop_mask <- mask(raster_choose_dem_crop, bound)

name <- paste("Heihe-dem", ".tif",sep="")

writeRaster(raster_choose_dem_crop_mask, name, format = 'GTiff', overwrite = TRUE)

dem_raster_value <- as.data.frame(raster_choose_dem_crop_mask, xy = TRUE)

colnames(dem_raster_value) <- c("lon", "lat", "dem")

write.table(dem_raster_value, "Yellow-dem-02.csv", row.names=FALSE, col.names=TRUE, sep=" ")

runoff_dem <- cbind(runoff_raster_value[ ,c("lon", "lat", "runoff")], dem_raster_value$dem)

colnames(runoff_dem) <- c("lon", "lat", "runoff", "dem")

runoff_dem_nodata <-na.omit(runoff_dem)

write.table(runoff_dem_nodata,"Yellow-runoff_dem_nodata-03.csv",row.names=FALSE, col.names=TRUE, sep=" ")

runoff_dem_nodata_order <- runoff_dem_nodata[order(runoff_dem_nodata$dem), ]

write.table(runoff_dem_nodata_order,"Yellow-runoff_dem_nodata_order-04.csv", row.names=FALSE, col.names=TRUE, sep=" ")

length <- length(runoff_dem_nodata_order$runoff)

length <- ceiling(length*0.02)

runoff_dem_nodata_order$dem <- filter(runoff_dem_nodata_order$dem/length, rep(1,length))

runoff_dem_nodata_order$runoff <- filter(runoff_dem_nodata_order$runoff/length, rep(1,length))

final_runoff_dem <- cbind(runoff_dem_nodata_order$dem, runoff_dem_nodata_order$runoff)

final_runoff_dem_nodata <-na.omit(final_runoff_dem)

colnames(final_runoff_dem_nodata) <- c("dem", "runoff")

write.table(final_runoff_dem_nodata,"Yellow-final_runoff_dem_nodata-05.csv", row.names=FALSE, col.names=FALSE, sep=" ")

fig <- data.frame(final_runoff_dem_nodata)

class(fig)

plot(fig$dem, fig$runoff)

## Part 3.4: Application example of hydrometeorology.

The calculation methodology for both the runoff coefficient and evapotranspiration coefficient remains consistent. In this case, the calculation process for the runoff coefficient is used as an illustration.

year<-rep(1998:2017)

###***1. Process TPFMD precipitation.***

setwd("‘home\\08-Yellow-runoff and evap coefficient-process\\Yellow-runoff-year")

bound<-raster("C:/Users/fanxi/Desktop/08-Yellow-runoff and evap coefficient-process/Yellow.tif")

setwd("‘home\\08-Yellow-runoff and evap coefficient-process\\TPFMD-1998-2017-year-tif-seven headwaters")

for (i in 1:length(year)){

year_name = year[i]

setwd("‘home\\08-Yellow-runoff and evap coefficient-process\\TPFMD-1998-2017-year-tif-seven headwaters")

name_select <- paste(year_name, "-TPFMD-prec.tif", sep="")

raster_choose <- raster(name_select)

raster_choose_resample <- resample(raster_choose, bound, method = "bilinear")

raster_choose_resample_mask <- mask(raster_choose_resample, bound)

name <- paste(year_name, "-Yellow_pre", ".tif",sep="")

setwd("‘home\\08-Yellow-runoff and evap coefficient-process\\Yellow-pre-year")

writeRaster(raster_choose_resample_mask, name, format = 'GTiff', overwrite = TRUE)

}

###***2. Calculate mean precipitation on the year scale.***

setwd("‘home\\08-Yellow-runoff and evap coefficient-process\\Yellow-pre-year")

name_select <- paste(".", "*", ".", "tif",sep="")

raster_pre_year <- stack(list.files(pattern = name_select))

raster_pre_year_mean <- mean(raster_pre_year, na.rm=TRUE)

plot(raster_pre_year_mean)

setwd("‘home\\08-Yellow-runoff and evap coefficient-process")

writeRaster(raster_pre_year_mean, "000-Yellow_pre.1998~2017_year_mean", format = 'GTiff', overwrite = TRUE)

###***3. Calculate runoff coefficient.***

for (i in 1:length(year)){

year_name = year[i]

setwd("‘home\\08-Yellow-runoff and evap coefficient-process\\Yellow-runoff-year")

runoff_name_select <- paste(year_name, "-Yellow_runoff", ".tif",sep="")

runoff_raster_choose <- raster(runoff_name_select)

setwd("‘home\\08-Yellow-runoff and evap coefficient-process\\Yellow-pre-year")

pre_name_select <- paste(year_name, "-Yellow_pre.tif", sep="")

pre_raster_choose <- raster(pre_name_select)

runoff_coefficient <- runoff_raster_choose/pre_raster_choose

runoff_coefficient_name <- paste(year_name, "-Yellow_runoff coefficient", ".tif",sep="")

setwd("‘home\\08-Yellow-runoff and evap coefficient-process\\Yellow-runoff coefficient-year")

writeRaster(runoff_coefficient, runoff_coefficient_name, format = 'GTiff', overwrite = TRUE)

}

###***4. Calculate mean runoff coefficient on the year scale.***

setwd("‘home\\08-Yellow-runoff and evap coefficient-process\\Yellow-runoff coefficient-year")

name_select <- paste(".", "*", ".", "tif",sep="")

raster_rc_year <- stack(list.files(pattern = name_select))

raster_rc_year_mean <- mean(raster_rc_year, na.rm=TRUE)

plot(raster_rc_year_mean)

setwd("‘home\\08-Yellow-runoff and evap coefficient-process")

writeRaster(raster_rc_year_mean, "000-Yellow_runoff_coefficient.1998~2017_year_mean", format = 'GTiff', overwrite = TRUE)

###***5. Calculate trend of runoff coefficient.***

library(broom)

setwd("‘home\\08-Yellow-runoff and evap coefficient-process\\Yellow-runoff coefficient-year")

name_select <- paste("^", ".", "*", ".", "tif", "$", sep="")

raster_rc <- stack(list.files(pattern = name_select))

time<-1:nlayers(raster_rc)

fun1 <- function(x) { if (is.na(x[1])){ NA } else lm(x ~ time)$coefficients[2] }

fun2 <- function(x) { if (is.na(x[1])){ NA } else glance(lm(x ~ time))$p.value }

fun3<-function(x) { if (is.na(x[1])){ NA } else glance(lm(x ~ time))$r.squared }

rc.b<-calc(raster_rc,fun1)

rc.p<-calc(raster_rc,fun2)

rc.r2<-calc(raster_rc,fun3)

raster_value <- as.data.frame(rc.b, xy = TRUE)

head(raster_value)

colnames(raster_value) <- c("lon", "lat", "data")

library(data.table)

raster_value <- as.data.table(raster_value)

raster_value[data == 0, data:= NA]

values(rc.b) <- c(raster_value$data)

rc.b

raster_value <- as.data.frame(rc.p, xy = TRUE)

head(raster_value)

colnames(raster_value) <- c("lon", "lat", "data")

library(data.table)

raster_value <- as.data.table(raster_value)

raster_value[data == 0, data:= NA]

values(rc.p) <- c(raster_value$data)

rc.p

setwd("‘home\\08-Yellow-runoff and evap coefficient-process")

writeRaster(rc.b, "000-Yellow_runoff_coefficient.1998~2017_year_trend_b", format = 'GTiff', overwrite = TRUE)

writeRaster(rc.p, "000-Yellow_runoff_coefficient.1998~2017_year_trend_p", format = 'GTiff', overwrite = TRUE)

## Part 3.5: Application example of carbon transport.

The procedure for determining the mean and trend is akin to the aforementioned process; hence, it will not be reiterated.

###***Calculate annual DOC.***

setwd("‘home\\10-calculate_C")

year<-rep(1998:2017)

slope <- raster("Yellow_Slope.tif")

for (i in 1:length(year)){

year_name = year[i]

setwd("‘home\\10-calculate_C\\Yellow-runoff-year")

name_runoff <- paste(year_name, "-Yellow_runoff.tif", sep="")

raster_runoff <- raster(name_runoff)

setwd("‘home\\10-calculate_C\\Yellow-GPP-year")

name_GPP <- paste(year_name, "-Yellow-GPP.tif", sep="")

raster_GPP <- raster(name_GPP)

raster_1 <- raster_GPP/raster_GPP

setwd("‘home\\10-calculate_C\\Yellow-C-year")

raster_C <- (raster_runoff*0.004 - slope*(pi/180)*8.76 + raster_runoff*0.095)*raster_1

raster_value <- as.data.frame(raster_C, xy = TRUE)

colnames(raster_value) <- c("lon", "lat", "data")

raster_value <- as.data.table(raster_value)

raster_value[data <= 0, data:= NA]

values(raster_C) <- c(raster_value$data)

name_C <- paste(year_name, "-Yellow", "-C", ".tif",sep="")

writeRaster(raster_C, name_C, format = 'GTiff', overwrite = TRUE)

}

###***Calculate annual WUE.***

setwd("‘home\\11-calculate_WUE")

year<-rep(1998:2017)

for (i in 1:length(year)){

year_name = year[i]

setwd("‘home\\11-calculate_WUE\\Yellow-evap-year")

name_evap <- paste(year_name, "-Yellow_evap.tif", sep="")

raster_evap <- raster(name_evap)

setwd("‘home\\11-calculate_WUE\\Yellow-GPP-year")

name_GPP <- paste(year_name, "-Yellow-GPP.tif", sep="")

raster_GPP <- raster(name_GPP)

raster_1 <- raster_GPP/raster_GPP

setwd("‘home\\11-calculate_WUE\\Yellow-WUE-year")

raster_WUE <- raster_GPP/raster_evap

raster_value <- as.data.frame(raster_WUE, xy = TRUE)

colnames(raster_value) <- c("lon", "lat", "data")

raster_value <- as.data.table(raster_value)

raster_value[data <= 0, data:= NA]

values(raster_WUE) <- c(raster_value$data)

name_WUE <- paste(year_name, "-Yellow", "-WUE", ".tif",sep="")

writeRaster(raster_WUE, name_WUE, format = 'GTiff', overwrite = TRUE)

}

## Part 3.6: Spatial correlation analysis of runoff and precipitation.

setwd("‘home\\07-CC of TPFMD_Pre and TPRED_Runoff\\Pre_TPFMD_Yellow_year_resample")

raster_pre<-rast(list.files(pattern='*.tif$'))

setwd("‘home\\07-CC of TPFMD_Pre and TPRED_Runoff\\Runoff_WEBDHM_Yellow_year")

raster_runoff<-rast(list.files(pattern='*.tif$'))

z <- c(raster_pre, raster_runoff)

fun_cor = function(x) {

Rs = Hmisc::rcorr(x[1:20], x[21:40], type = "spearman")

Rx = Rs$r[2]

Px = Rs$P[2]

return(c(Rx, Px))

}

r_pre_runoff = app(z,fun_cor,cores=4)

names(r_pre_runoff) <- c("coefficient","p_value")

plot(r_pre_runoff)

r <- subset(r_pre_runoff,1)

p <- subset(r_pre_runoff,2)

writeRaster(r, filename = "‘home\\07-CC of TPFMD_Pre and TPRED_Runoff\\r_pre_runoff_Yellow.tif", overwrite = TRUE)

writeRaster(p, filename = "‘home\\07-CC of TPFMD_Pre and TPRED_Runoff\\p_pre_runoff_Yellow.tif", overwrite = TRUE)

# Part 4: References

1. Ye, Q. *et al.* Glacier changes on the Tibetan Plateau derived from Landsat imagery: Mid-1970s - 2000-13. *J. Glaciol.* **63**, 273–287 (2017).

2. Guo, W. *et al.* The second Chinese glacier inventory: Data, methods and results. *J. Glaciol.* **61**, 357–372 (2015).

3. Wang, Y. *et al.* Vanishing Glaciers at Southeast Tibetan Plateau Have Not Offset the Declining Runoff at Yarlung Zangbo. *Geophys. Res. Lett.* **48**, 1–12 (2021).

4. Wang, Y. *et al.* Impacts of frozen ground degradation and vegetation greening on upper Brahmaputra runoff during 1981–2019. *Int. J. Climatol.* **43**, 3768–3781 (2023).

5. Liu, H. *et al.* Energy-balance modeling of heterogeneous glacio-hydrological regimes at upper Indus. *J. Hydrol. Reg. Stud.* **49**, 101515 (2023).

6. Zhao, X. *et al.* The global land surface satellite (GLASS) remote sensing data processing system and products. *Remote Sens.* **5**, 2436–2450 (2013).

7. He, J. *et al.* The first high-resolution meteorological forcing dataset for land process studies over China. *Sci. Data* **7**, 1–11 (2020).

8. Jiang, Y. et al. TPHiPr: a long-term (1979-2020) high-accuracy precipitation dataset (1/30°, daily) for the Third Pole region based on high-resolution atmospheric modeling and dense observations. *Earth Syst. Sci. Data* **15**, 621–638 (2023).

9. Hersbach, H. *et al.* The ERA5 global reanalysis. *Q. J. R. Meteorol. Soc.* **146**, 1999–2049 (2020).

10. Muñoz-Sabater, J. *et al.* ERA5-Land: A state-of-the-art global reanalysis dataset for land applications. *Earth Syst. Sci. Data* **13**, 4349–4383 (2021).

11. Qi, W., Zhang, C., Fu, G. & Zhou, H. Global Land Data Assimilation System data assessment using a distributed biosphere hydrological model. *J. Hydrol.* **528**, 652–667 (2015).

12. Wang, Y., Wang, L., Li, X., Zhou, J. & Hu, Z. An integration of gauge, satellite, and reanalysis precipitation datasets for the largest river basin of the Tibetan Plateau. *Earth Syst. Sci. Data* **12**, 1789–1803 (2020).

13. Wang, L. *et al.* TP-river monitoring and quantifying total river runoff from the third pole. *Bull. Am. Meteorol. Soc.* **102**, E948–E965 (2021).

14. Song, L., Wang, L., Luo, D., Chen, D. & Zhou, J. Assessing hydrothermal changes in the upper Yellow River Basin amidst permafrost degradation. *npj Clim. Atmos. Sci.* **7**, 1–12 (2024).

15. Song, L. *et al.* Improving Permafrost Physics in a Distributed Cryosphere-Hydrology Model and Its Evaluations at the Upper Yellow River Basin. *J. Geophys. Res. Atmos.* **125**, 1–22 (2020).

16. Wan, Z. New refinements and validation of the collection-6 MODIS land-surface temperature/emissivity product. *Remote Sens. Environ.* **140**, 36–45 (2014).

17. Muhammad, S. & Thapa, A. Daily Terra-Aqua MODIS cloud-free snow and Randolph Glacier Inventory 6.0 combined product (M∗D10A1GL06) for high-mountain Asia between 2002 and 2019. *Earth Syst. Sci. Data* **13**, 767–776 (2021).

18. Engelhardt, M. *et al.* Modelling 60 years of glacier mass balance and runoff for Chhota Shigri Glacier, Western Himalaya, Northern India. *J. Glaciol.* **63**, 618–628 (2017).

19. Ghiggi, G., Humphrey, V., Seneviratne, S. I. & Gudmundsson, L. GRUN: An observation-based global gridded runoff dataset from 1902 to 2014. *Earth Syst. Sci. Data* **11**, 1655–1674 (2019).

20. Gou, J., Miao, C., Samaniego, L., Xiao, M., Wu, J. & Guo, X. CNRD v1.0: a high‒quality natural runoff dataset for hydrological and climate studies in China. *Bull. Am. Meteorol. Soc.* **5**, E929–E947. (2021).

21. Alfieri, L. *et al.* A global streamflow reanalysis for 1980–2018. *J. Hydrol. X* **6**, 100049 (2020).

22. Suzuki, T. *et al.* A dataset of continental river discharge based on JRA-55 for use in a global ocean circulation model. *J. Oceanogr.* **74**, 421–429 (2018).

23. Lin, P. *et al.* Global Reconstruction of Naturalized River Flows at 2.94 Million Reaches. *Water Resour. Res.* **55**, 6499–6516 (2019).

24. Yang, Y. *et al.* Global reach-level 3-hourly river flood reanalysis (1980–2019). *Bull. Am. Meteorol. Soc.* **102**, E2086–E2105 (2021).

25. Han, C. *et al.* Long-term variations in actual evapotranspiration over the Tibetan Plateau. *Earth Syst. Sci. Data* **13**, 3513–3524 (2021).

26. Song, L., Zhuang, Q., Yin, Y., Zhu, X. & Wu, S. Spatio-temporal dynamics of evapotranspiration on the Tibetan Plateau from 2000 to 2010 Spatio-temporal dynamics of evapotranspiration on the Tibetan Plateau from 2000 to 2010. *Environ. Res. Lett.* **12**, 014011 (2017).

27. Zheng, C., Jia, L. & Hu, G. Global land surface evapotranspiration monitoring by ETMonitor model driven by multi-source satellite earth observations. *J. Hydrol.* **613**, 128444 (2022).

28. Zhang, K., Zhu, G., Ma, N., Chen, H. & Shang, S. Improvement of evapotranspiration simulation in a physically based ecohydrological model for the groundwater–soil–plant–atmosphere continuum. *J. Hydrol.* **613**, (2022).

29. Fu, J. *et al.* Improved global evapotranspiration estimates using proportionality hypothesis-based water balance constraints. *Remote Sens. Environ.* **279**, 113140 (2022).

30. Lu, J. *et al.* A harmonized global land evaporation dataset from model-based products covering 1980-2017. *Earth Syst. Sci. Data* **13**, 5879–5898 (2021).

31. Li, C. *et al.* CAMELE: Collocation-Analyzed Multi-source Ensembled Land Evapotranspiration Data. *Eart. Syst. Sci. Data* **16**, 1811–1846 (2022).

32. Feng, Q. *et al.* Long-term gridded land evapotranspiration reconstruction using Deep Forest with high generalizability. *Sci. Data* **10**, 1–13 (2023).

33. Martens, B. *et al.* GLEAM v3: Satellite-based land evaporation and root-zone soil moisture. *Geosci. Model Dev.* **10**, 1903–1925 (2017).

34. Matveeva, T. & Sidorchuk, A. Modelling of surface runoff on the Yamal Peninsula, Russia, using ERA5 reanalysis. *Water (Switzerland)* **12**, (2020).

35. Xu, C., Wang, W., Hu, Y. & Liu, Y. Evaluation of ERA5, ERA5-Land, GLDAS-2.1, and GLEAM potential evapotranspiration data over mainland China. *J. Hydrol. Reg. Stud.* **51**, 101651 (2024).

36. Chen, J. *et al.* Basin-Scale River Runoff Estimation From GRACE Gravity Satellites, Climate Models, and In Situ Observations: A Case Study in the Amazon Basin. *Water Resour. Res.* **56**, 1–21 (2020).

37. Bell, B. *et al.* The ERA5 global reanalysis: Preliminary extension to 1950. *Q. J. R. Meteorol. Soc.* **147**, 4186–4227 (2021).

38. Albergel, C., Balsamo, G., De Rosnay, P., Muñoz-Sabater, J. & Boussetta, S. A bare ground evaporation revision in the ECMWF land-surface scheme: Evaluation of its impact using ground soil moisture and satellite microwave data. *Hydrol. Earth Syst. Sci.* **16**, 3607–3620 (2012).

39. Bain, R. L. *et al.* Intercomparison of global ERA reanalysis products for streamflow simulations at the high-resolution continental scale. *J. Hydrol.* **616**, 128624 (2023).

40. Zsoter, E. *et al.* How well do operational numerical weather prediction configurations represent hydrology? *J. Hydrometeorol.* **20**, 1533–1552 (2019).

41. Nie, Y. *et al.* Glacial change and hydrological implications in the Himalaya and Karakoram. *Nat. Rev. Earth Environ.* **2**, 91–106 (2021).

42. Kirillin, G. *et al.* Physics of seasonally ice-covered lakes: A review. *Aquat. Sci.* **74**, 659–682 (2012).

43. Wang, L. *et al.* Modeling Glacio-Hydrological Processes in the Himalayas: A Review and Future Perspectives. *Geogr. Sustain.* **5**, 179–192 (2024).

44. Ragettli, S., Immerzeel, W. W. & Pellicciotti, F. Contrasting climate change impact on river flows from high-altitude catchments in the Himalayan and Andes Mountains. *Proc. Natl. Acad. Sci. U. S. A.* **113**, 9222–9227 (2016).

45. Wang, L. *et al.* Development of a distributed biosphere hydrological model and its evaluation with the Southern Great Plains experiments (SGP97 and SGP99). *J. Geophys. Res. Atmos.* **114**, 1–15 (2009).

46. Wang, L. *et al.* Development of a land surface model with coupled snow and frozen soil physics. *Water Resour. Res.* **53**, 5085–5103 (2017).
